# Metabolites with a message: impacts on epigenetics and implications for epimetabopathies

**DOI:** 10.1038/s44319-026-00891-5

**Published:** 2026-07-29

**Authors:** Indrakshi Banerjee, Atanu Mondal, Siddhesh S Kamat, Chandrima Das

**Affiliations:** 1 Biophysical Sciences Division, Saha Institute of Nuclear Physics, Kolkata, India; 2 Homi Bhabha National Institute, Training School Complex, Mumbai, India; 3 Department of Biology, Indian Institute of Science Education and Research, Pune, India

**Keywords:** Chromatin, Transcription & Genomics, Metabolism

## Abstract

Once identified primarily as a bioenergetic organelle, the mitochondrion has now emerged as a pivotal signalling hub that communicates with the nucleus to shape cellular fate. It integrates the cell’s metabolic state with transcriptional and epigenetic programs, tweaking gene expression. Mitochondrial metabolites serve as regulators of cellular physiology, functioning as important signalling intermediates and modulating enzymes involved in epigenetic modifications. In parallel, nuclear transcriptional programs govern mitochondrial biogenesis, dynamics and quality control to preserve metabolic homeostasis under stress. Moreover, circulating metabolites can function as systemic messengers coordinating interorgan crosstalk and immune responses. Perturbations in this dynamic reciprocity can rewire the cellular script and spiral into “epimetabopathies”, where metabolic-epigenetic conflicts ignite pathological conditions. This review discusses how mitochondria-nucleus crosstalk coordinates genome surveillance, metabolite-driven epigenetic regulation and systemic metabolic signalling. It further offers an overview of epimetabopathies with potential implications for future diagnostics and therapeutics.

## Abstract (web summary)

This review discusses how mitochondria-nucleus crosstalk coordinates genome surveillance, metabolite-driven epigenetic regulation and systemic metabolic signalling.

## Graphical abstract

**Figure:**
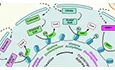

## Introduction

Every living cell is like a tiny battlefield, constantly defending itself against environmental and endogenous assaults. To thrive in such a challenging environment, maintaining metabolic homeostasis is of utmost importance. Metabolites are not just byproducts of complex biochemical reactions. They are messengers of cellular growth, adaptation and survival (Medina et al, 2020; Baker and Rutter, 2023; Ma et al, 2024; Mao et al, 2026). These molecular signals are like cellular currency exchanged between organelles, especially the mitochondria and the nucleus, trading vital information to maintain balance during stress. Mitochondria, long credited for fuelling the cell through oxidative phosphorylation, are much more than that. They are also regulators of the genome and the epigenome, safeguarding the cell’s genetic blueprint (Lombardi et al, 2019; Cao et al, 2022; Tatar and Sedivy, 2016). They play pivotal roles in regulating the supply of deoxynucleotide triphosphate (dNTPs), indispensable for DNA replication and damage repair (Leanza et al, 2008). They also control the activity of several DNA repair enzymes (Fang et al, 2016; Wu et al, 2019). Genomic integrity is thus maintained by a complex, tripartite regulatory network that links mitochondrial metabolism, nuclear epigenetic modifications and DNA repair-replication processes. Mitochondrial tricarboxylic acid (TCA) cycle intermediates, like acetyl-CoA and α-ketoglutarate, serve as essential substrates for histone acetyltransferases and demethylases, respectively. This directly couples cellular energetic status to chromatin accessibility and gene expression. One-carbon and fatty acid metabolism also play a significant role in shaping the epigenome. The idea of reciprocal signalling, where the nucleus and mitochondria constantly exchange messages, unravels a whole new frontier in organellar crosstalk (Quirós et al, 2016). Here, we explore how fleeting mitochondrial metabolites are converted into persistent epigenetic alterations that underpin cellular resilience. Consequently, dysfunctions in the intertwined mito-nuclear dialogue can cause “epimetabopathies”, where metabolic derangements can fuel epigenetic disorders and vice versa.

## Mitochondria in genome surveillance, beyond powerhouse duties

Over a billion years ago, a dramatic cellular transformation resulted from an alpha-proteobacterium entering a proto-eukaryotic host cell and sparking the origin of mitochondria (Yang et al, 1985; Wallace, 1999; Lane and Martin, 2010). This endosymbiotic partnership necessitated the coordinated regulation of the two evolutionarily distinct mitochondrial DNA (mtDNA) and nuclear DNA (nDNA) to maintain cellular function. Leaving a nominal mtDNA required for encoding the essential OXPHOS subunits, most of the genes essential for mitochondrial biogenesis and functioning got integrated into the chromosomal DNA (Patananan et al, 2016; Wallace, 2005). Eukaryotic cells thus evolved a precise intergenomic regulatory mechanism involving transcriptional and translational crosstalk between these two subcellular organelles, which thereby regulate the metabolic milieu. From an evolutionary perspective, the continuous mito-nuclear coevolution gave rise to a metabolite-driven signalling to allow functional harmony. Thus, apart from operating as the cellular ATP factory, the mitochondria play a pivotal role in communicating stress and metabolic cues to the nucleus, regulating organismal health and ailment (Chandel, 2015; Nunnari and Suomalainen, 2012).

The mitochondria are the major hub of several metabolic pathways like fatty acid oxidation, amino acid metabolism, oxidative phosphorylation and the TCA cycle. In addition, they play a vital role in supporting nucleotide metabolism. While most nucleotide biosynthesis occurs in the cytosol and nucleus, in the absence of mitochondria, the overall balance in nucleotide pools is severely compromised (Desler et al, 2007, 2010).

### Mitochondrial governance of dNTP supply and genome stability

The mitochondria have transcended their canonical role in energy metabolism and emerged as efficient regulators of genomic integrity. Rather than functioning as secluded entities, they dynamically regulate the cytosolic nucleotide pool. Likewise, the cytosolic nucleotide availability affects mtDNA replication. An imbalance in this exchange can cause genomic instability in both nDNA and mtDNA (Pontarin et al, 2003; Song et al, 2003; Awoyomi et al, 2025; Desler et al, 2007). The mitochondria control the cellular dNTP reservoir by coupling oxidative metabolism with nucleotide biosynthesis pathways. For instance, dihydroorotate dehydrogenase (DHODH), an inner mitochondrial membrane enzyme, plays a fundamental role in the de novo pyrimidine synthesis pathway by converting dihydroorotate (DHO) to orotate (Rawls et al, 2000; Evans and Guy, 2004; Löffler et al, 1997). DHODH banks on ubiquinone as an electron acceptor, thus integrating the mitochondrial respiratory chain with nucleotide biosynthesis. Depletion of DHODH results in impaired mitochondrial respiration, reduced OXPHOS efficiency and decreased mitochondrial membrane potential. This triggers the generation of reactive oxygen species (ROS) and subsequent mitochondrial dysfunction (Fang et al, 2013). Damage to the electron transport chain further leads to an imbalance in the cytosolic dNTP pools as a result of DHODH inhibition (Singh, 2004; Desler et al, 2010). Other than biosynthesis, mitochondria seem to selectively sequester certain species of dNTP, particularly guanine deoxynucleotide (dGTP), acting like nucleotide reservoirs. The mitochondrial dGTP supply happens to be securely bound to the NDUFA10 subunit of respiratory complex I, demonstrating a structural link between genome maintenance and the OXPHOS complex (Molina-Granada et al, 2022). The electron transport chain also plays an indispensable role in the biosynthesis of aspartate, an essential precursor for de novo pyrimidine synthesis. Respiratory impairment thus restricts dNTP availability, a defect that can be rescued by exogenous aspartate. This directly couples redox metabolism to DNA replication efficiency and places mitochondrial respiration as an upstream determining factor of dNTP pool growth (Sullivan et al, 2015; Birsoy et al, 2015). Acute stress triggered by mitochondrial singlet-oxygen species produced during respiration can trigger mitoROS-dependent telomeric double-strand breaks. Telomeres, being repetitive G-rich regions, are extremely sensitive to dNTP pool imbalance and oxidative stress-induced nucleotide damage. Telomeres may therefore be thought to serve as early nuclear sensors of metabolic and redox distress in the mitochondria (Qian et al, 2019; Wu et al, 2021).

Further insight into the synchronised regulation of nDNA and mtDNA integrity has emerged in the context of dGTP metabolism. This metabolic interdependence has been substantiated by the fact that perturbation in one cellular compartment can reform dGTP availability in the other, thereby coordinating replication fidelity across genomes (Leanza et al, 2008). Interestingly, mtDNA replication defects can “rob” the nucleus of the cytosolic dNTP pool, instigating a threat to nDNA stability (Hämäläinen et al, 2019). Due to this sequestration, there is a resultant scarcity of dNTPs in the nucleus, which causes replication fork stalling and breaks in the nDNA. Moreover, shortcomings in mtDNA replication due to TWINKLE helicase mutations perturb the total cellular dNTP pool. This induces adaptive remodelling of folate-mediated one-carbon metabolism (Nikkanen et al, 2016). In addition to their canonical role in aspartate and one-carbon metabolism, mitochondria regulate nuclear genome integrity through the biogenesis of iron–sulphur (Fe–S) clusters. Defects in this pathway can lead to genomic instability due to cofactor deficiency (Veatch et al, 2009).

Taken together, these findings suggest that compromised mitochondrial functioning fosters skewed dNTP ratios, misincorporation by polymerases, replicative stress and chromosomal instability, augmenting mutational burden.

### Tuning of DNA repair enzymes by mitochondria

When stressed with genotoxic agents, our cells employ a remarkable defence system known as the DNA damage response (DDR) (Zhou and Elledge, 2000; Jackson and Bartek, 2009). Like an elite emergency squad, the DDR rapidly detects the site of injury, halts the cell cycle to prevent further mistakes and recruits an arsenal of repair enzymes to fix the problem. Such enzymes are dynamically tweaked by the mitochondria by integrating cellular metabolism with genome maintenance.

Emerging evidence positions mitochondria as upstream regulators of the repair processes by coupling cellular metabolic state to nuclear genome maintenance. For example, during oxidative phosphorylation, mitochondria generate ATP along with the potent damaging agents ROS, thereby influencing DNA integrity and the energetic and redox conditions required for DNA repair (Nissanka and Moraes, 2018; Scheibye-Knudsen et al, 2015). It has been observed that impaired mitochondrial function compromises both nuclear genome stability and the DDR through depletion of metabolic cofactors, like ATP, acetyl-CoA, α-ketoglutarate and NAD^+^, essential for repair enzyme function (Nadalutti et al, 2022; Sykora et al, 2017; Hou et al, 2018). For example, the energy-intensive DNA repair processes and chromatin remodelling rely heavily on ATP availability. Metabolites like acetyl-CoA and α-ketoglutarate regulate chromatin accessibility and epigenetic modifications, thereby influencing DNA repair efficiency, as discussed in subsequent sections. The NAD^+^ pool of the cell serves as a central node connecting mitochondrial redox state to both Poly (ADP-ribose) polymerase (PARP) -mediated DNA repair and sirtuin-dependent epigenetic regulation (Li et al, 2017; Lehmann et al, 2016; Croteau et al, 2017). The NAD^+^-dependent deacetylase, SIRT1, can be identified as a metabolic-genomic rheostat that links mitochondrial functioning to nDNA repair. SIRT1 drives both mitochondrial biogenesis and enhanced DNA repair by Ku70 deacetylation, aiding non-homologous end joining of double-strand breaks. The mito-nuclear control of genome maintenance is thus reinforced by SIRT1 through its coordinated regulation of mitochondrial homeostasis and repair of nuclear double-strand DNA breaks (Majeed et al, 2021; Jeong et al, 2007).

Furthermore, mitochondria are involved in several DNA repair disorders like ataxia-telangiectasia (AT), Cockayne syndrome (CS) and xeroderma pigmentosum (XP), to name a few (Scheibye-Knudsen et al, 2012; Valentin-Vega et al, 2012; Shiloh and Ziv, 2013; Fang et al, 2016). The disruption of mitochondrial quality control severely impairs DNA repair capacity in xeroderma pigmentosum group A (XPA), a nucleotide excision repair disease. A key mechanism linking mitochondria to DDR regulation can be exemplified by the NAD^+^-SIRT1-PGC-1α axis. In XPA cells, persistent DNA damage leads to the chronic hyperactivation of PARP-1, a crucial NAD⁺-consuming enzyme involved in DDR. Depletion in NAD^+^ results in impaired SIRT1-dependent deacetylation, attenuating the downstream Peroxisome proliferator-activated receptor gamma co-activator 1-alpha (PGC-1α) pathway, ultimately compromising DNA repair capacity in XPA cells. This decreased activity of the NAD^+^-SIRT1-PGC-1α axis can be corrected by supplementation with NAD^+^ precursors (Fang et al, 2014; Verdin, 2015; Cantó et al, 2015; Bai et al, 2011).

Likewise, mitochondrial stress influences the mito-nuclear communication, explicitly linking this to DNA repair processes. mtDNA stressors and inhibitors of mitochondrial metabolism trigger retrograde calcium signalling (Biswas et al, 1999). The released Ca^2+^ ions from the mitochondria result in reduced activity of nuclear factor-κB (NF-κB), a factor directly linked to DDR and post-translational modification of key repair proteins (Biswas et al, 1999; McCool and Miyamoto, 2012; Janssens and Tschopp, 2006). These findings, linking mtDNA stress and nuclear transcription, indicate that mitochondrial dysfunction, through disrupted Ca²⁺ signalling, can influence the efficiency of DDR mechanisms.

Notably, mitochondria, the major producers of cellular ATP, fine-tune replication fidelity and resilience to stress via spatiotemporally controlled energy relocation. Mitochondrial positioning can regulate local ATP availability near the nucleus, particularly under mechanical confinement, where increased nucleus-mitochondria proximity generates a localised nuclear ATP surge that supports efficient repair and cell cycle progression (Ghose et al, 2025). Thus, spatial redistribution of mitochondria offers an additional layer of control over nuclear repair dynamics by modulating energy supply.

The mitochondrial structure and function are other critical determinants of the nuclear DDR. Genotoxic assault usually promotes transient mitochondrial hyperfusion to mitigate the stress. Loss of fusion by MFN1/MFN2 disruption results in fragmented mitochondria, dampened ATM signalling and failure in the activation of the JNK signalling pathway. Following this, there is a reduction in BRCA1 and 53BP1 foci formation, compromising both homologous recombination and non-homologous end-joining mediated DNA damage repair (Oanh et al, 2022).

Collectively, these findings support a model in which mitochondrial metabolic capacity, signalling, spatial organisation, morphology and dynamics act in concert to regulate DDR efficiency through coordinated control of cellular energy state, redox balance and activation of DNA repair pathways.

## Reciprocal rewiring of chromatin and mitochondria

The orchestrated mito-nuclear communication operates bidirectionally via intricate anterograde and retrograde signalling, essential for integrating the metabolic and genomic demands of the cell. The nuclear anterograde signalling exerts mitochondrial control by regulating nuclear-encoded mitochondrial gene expression. Conversely, the mitochondria relay information back to the nucleus through retrograde signals to regulate transcriptional and epigenetic reprogramming. The persistent molecular crosstalk between these two complementary axes is essential for maintaining organellar health (Mottis et al, 2019; Hino et al, 2022), and perturbations result in the pathogenesis of a wide spectrum of diseases.

### Chromatin shapes mitochondrial yield: the anterograde control

**Figure 1:**
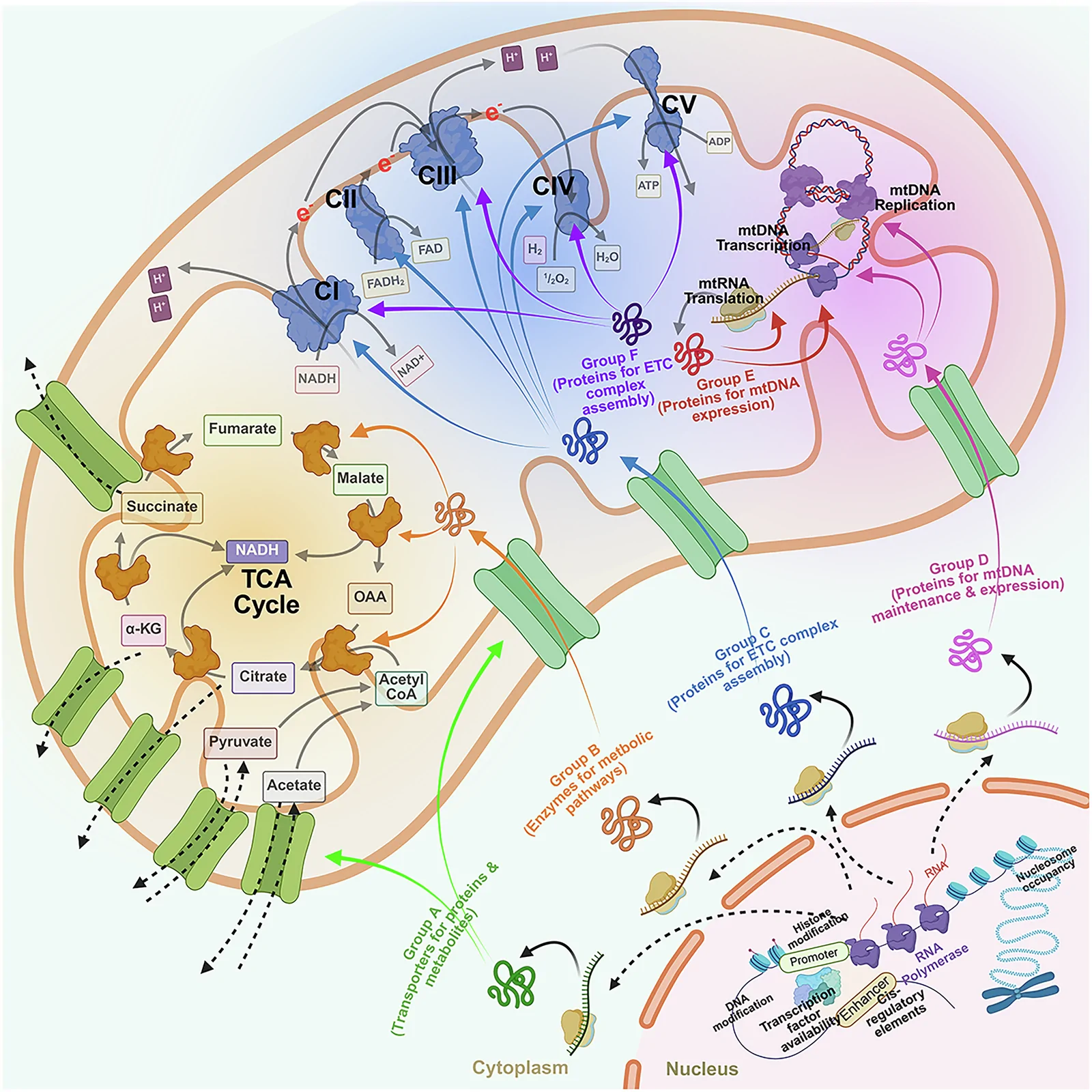
Nuclear regulation of mitochondrial morphology and function. Schematic overview of the anterograde signalling pathway, highlighting nuclear control of mitochondrial metabolism, respiratory chain complex assembly and mtDNA expression. Nuclear transcription is regulated by chromatin architecture, histone modifications, transcription factor availability, enhancer–promoter interactions and RNA polymerase recruitment. The transcribed mRNAs are translated in the cytosol to produce distinct classes of nuclear-encoded mitochondrial proteins. Group A (green): Membrane transporters and metabolite carriers that mediate the exchange of substrates and cofactors across mitochondrial membranes. Group B (orange): Enzymes of the tricarboxylic acid (TCA) cycle and associated mitochondrial metabolic pathways. Group C (blue): Structural subunits and assembly factors necessary for the electron transport chain (ETC) complexes. Group D (pink): Proteins involved in mtDNA maintenance, replication and genome stability. These factors regulate the production of two classes of mitochondria-encoded proteins, namely Group E and Group F. Group E (red): Proteins required for mtDNA transcription, RNA processing and mitochondrial translation and Group F (purple): mtDNA-encoded polypeptides are synthesised within the mitochondria to complete the structural framework of the ETC. Within the mitochondria, reducing equivalents (NADH and FADH₂) generated by the TCA cycle donate electrons to the ETC complexes I–IV (CI–CIV), driving proton pumping across the inner mitochondrial membrane to sustain oxidative phosphorylation. Arrows indicate metabolite flux, protein trafficking and regulatory direction. This integrated network highlights the multilayered nuclear control over mitochondrial bioenergetics, genome expression and metabolic plasticity. Created in BioRender. Das C (2026) https://BioRender.com/lcbt0w1.

The accessibility of chromatin is a fundamental determinant of transcription of genes controlling mitochondrial biogenesis and function (Fig. 1). A vast majority of mitochondrial proteins are encoded by the nucleus and influence mitochondrial function through two major mechanisms.

The first mechanism includes the nucleus-encoded metabolic enzymes, mitochondrial membrane transporters and quality-control proteins (Pfanner et al, 2019). The nuclear chromatin state directly or indirectly governs the mitochondrial metabolite levels of acetyl-CoA, α-ketoglutarate, NAD⁺ and S-adenosylmethionine, which afterwards serve as essential cofactors or donors for chromatin-modifying enzymes (Nadalutti et al, 2022). Similarly, the hepatic lysine demethylase LSD1 acts as an anterograde regulator of nuclear-encoded mitochondrial genes. By modifying histone methylation patterns, LSD1 licenses the expression of genes required for oxidative phosphorylation and mitochondrial biogenesis. Its loss compromises respiratory capacity and triggers a downstream stress response marked by elevated levels of FGF21, a hepatokine (Cao et al, 2021). Altogether, anterograde signalling places chromatin as a central orchestrator of mitochondrial homeostasis, yet this regulatory axis is not unidirectional.

The second mechanism comprises nuclear-encoded mitochondrial transcription factors and proteins to govern mtDNA replication. For example, the nuclear-encoded factors TFAM, TFB1M, TFB2M and mTERF are essential factors for mitochondrial genome expression. While TFAM and TFB2M are two crucial initiation factors, TFB1M enables proper mitochondrial ribosome assembly, and mTERF is the mitochondrial transcription termination factor (Litonin et al, 2010; Ramachandran et al, 2016; Koeck et al, 2011; Liu et al, 2019; Roberti et al, 2009). These proteins are produced in coordination with nucleus-encoded respiratory genes via signal-responsive transcriptional circuits. For instance, in the anterograde pathway, this synchronised production is achieved through certain nuclear transcription factors that regulate mitochondrial biogenesis. These transcription factors include the nuclear respiratory factors NRF-1 and NRF-2, PPAR (peroxisome proliferator-activated receptors), ERR (oestrogen-related receptors) and YY1 (ying yang 1) (Chau et al, 1992; Scarpulla, 2006; Liu et al, 2023; Blättler et al, 2012). The activities of these factors are further enhanced by the transcriptional co-activator family, PGC-1, comprising PGC-1α, PGC-1β, and PRC, which sense and adapt to variations in nutrient levels and the energetic needs of the cell (Cunningham et al, 2007; Finck and Kelly, 2006). Together, the transcription factors with their coactivators induce the expression of the Mitochondrial Transcription Factor A (TFAM), responsible for maintaining mtDNA stability and its gene expression (Larsson et al, 1998; Scarpulla, 2008). However, TFAM not only organises the mtDNA within nucleoids; it also functions in the cytosol to bind stress-released mtDNA to facilitate its clearing, thus dampening immune activation (Liu et al, 2024).

### Mitochondria rewrites chromatin: the retrograde feedback

Retrograde signalling, or the mitochondrial retrograde response (MRR), in particular, communicates mitochondrial stress to the nucleus, shaping the identity and function of metabolic tissues (Walker et al, 2025; Desai et al, 2020). Thus, signals from the mitochondria themselves can rewire the chromatin landscape to modulate transcription. For example, a mild respiratory chain malfunction causing mitochondrial proteostatic imbalance triggers large-scale chromatin reorganisation through higher H3K9 dimethylation. H3K9me2, conventionally a repressive mark, globally silences chromatin but selectively opens up specific loci to activate the mitochondrial unfolded protein response (UPRmt), permitting transcriptional activation of stress-responsive genes (Tian et al, 2016). Retrograde signalling alters epigenetic substrate availability by directly controlling the levels of essential chromatin-modifying metabolites, thereby tweaking transcriptional programs (Sahu and Lu, 2025; Etchegaray and Mostoslavsky, 2016). For example, the mitochondrial acetyl-CoA pool, serving as a substrate for histone acetyltransferases (HATs), impacts cellular gene expression patterns (Morrish et al, 2010). Other TCA cycle intermediates, like α-ketoglutarate, succinate and fumarate, modulate the activity of TET (Ten-Eleven Translocation) enzymes, i.e., dioxygenases that promote the removal of methyl marks from DNA (Laukka et al, 2016). Similarly, the critical TCA cycle electron acceptor and coenzyme, NAD⁺ is the fuel for sirtuin deacetylases and PARP enzymes involved in DDR (Verdin, 2015; Bai and Cantó, 2012; Imai and Guarente, 2014). Another critical signalling messenger, mitochondria-derived ROS, retro-communicates with the nucleus to activate certain redox-sensitive kinases and transcription factors for stress adaptation and antioxidant defence (Formentini et al, 2012; Sena and Chandel, 2012; Al-Mehdi et al, 2012; Bell et al, 2007). Further discussion of mitochondrial metabolites governing chromatin modifications will be in the next section.

Perturbations in mitochondria, like impaired calcium buffering, promote the nuclear translocation of NFAT, NF-κB and ATF transcription factors to restructure the gene regulatory landscape and compensate for the dysfunction (Butow and Avadhani, 2004; Biswas et al, 2003; Roy Chowdhury et al, 2020). Mitochondrial defects lead to integrated stress response activation, where eIF2α gets phosphorylated, dampening global translation but selectively enhancing ATF4-driven transcriptional programs to attain cellular equilibrium (Guo et al, 2020; Harding et al, 2003; Mick et al, 2020). Mitochondria are not merely metabolic engines, but dynamic signalling hubs that convey bioenergetic, redox and stress signals back to the nucleus for reconfiguring systemic homeostasis.

## Metabolites inscribe stress signals into the epigenome

**Figure 2:**
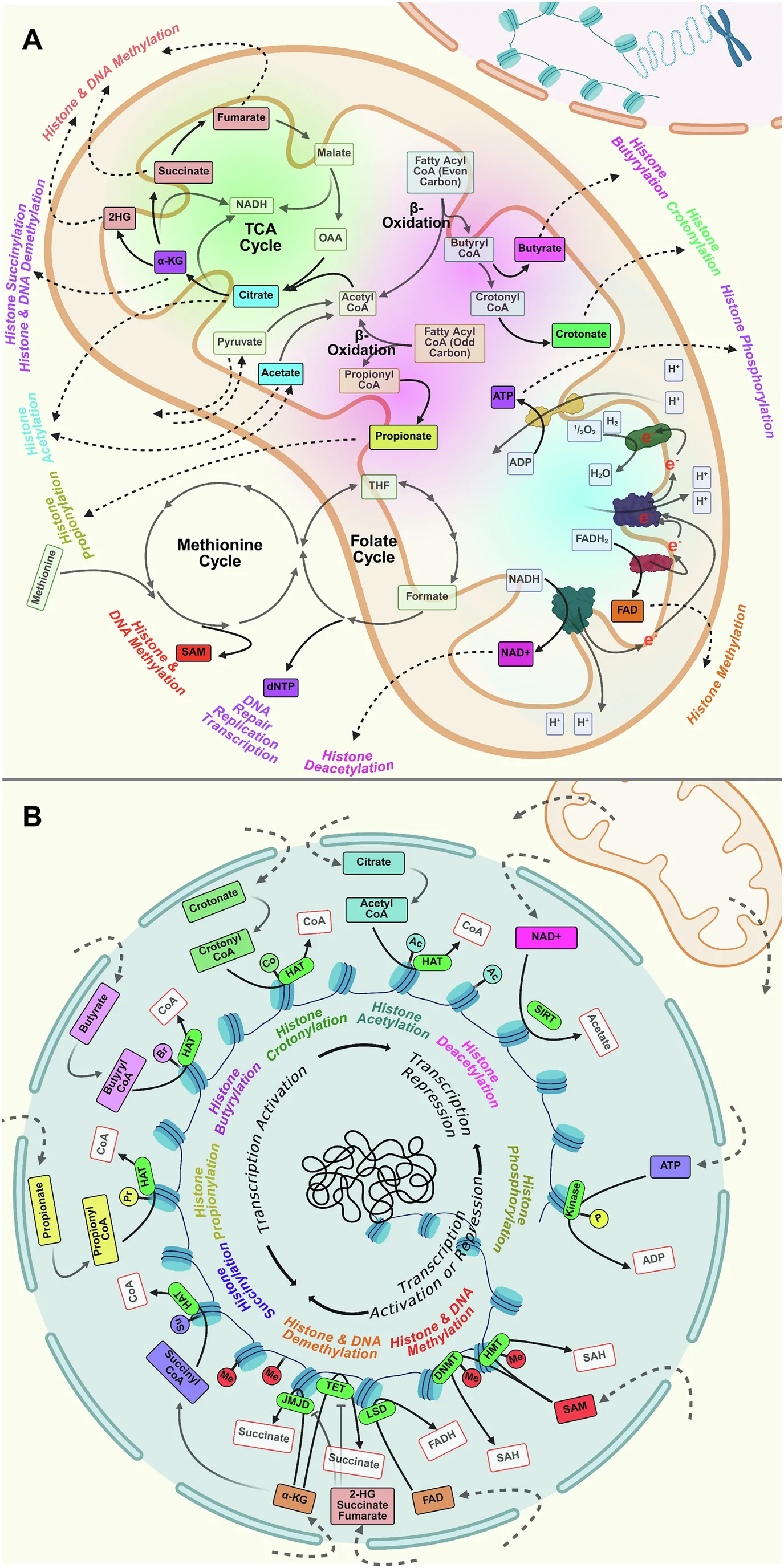
Mitochondrial metabolism-mediated regulation of nuclear gene expression. (**A**) Mitochondrial metabolic cues to the nucleus. The metabolites and intermediates produced by folate and methionine cycles (one-carbon metabolism), TCA cycle and β-oxidation are essential for communicating mitochondrial activity to the nucleus. The methionine and folate cycles form intertwined branches of one-carbon metabolism that couple cellular proliferation with epigenetic control. Folate-dependent one-carbon units feed the methionine cycle to generate S-adenosylmethionine (SAM), the principal methyl group donor for DNA and histone methylation. It also supports thymidylate and purine synthesis to maintain the deoxyribonucleotide (dNTP) pool required for DNA replication and repair. TCA cycle intermediates like citrate molecules are exported to the cytosol and converted into acetyl-CoA, supporting histone acetylation. α-Ketoglutarate (α-KG) is a crucial cofactor for α-KG-dependent dioxygenases, whereas 2-hydroxyglutarate (2HG), succinate and fumarate can accumulate and competitively inhibit these enzymes, thereby influencing DNA and histone methylation. Even- and odd-chain fatty acid oxidation produces short-chain acyl-CoAs like butyryl-, crotonyl- and propionyl-CoA, that serve as donors for corresponding histone acyl modifications. Oxidative phosphorylation supplies ATP and preserves redox balance by oxidising NADH and FADH₂ to regenerate NAD⁺ and FAD, which in turn regulate the acetylation landscape. Collectively, mitochondrial metabolites act as substrates, cofactors and metabolic cues that transmit organellar status to the nucleus. (**B**) Nuclear decoding of mitochondrial metabolites. Once transported to the nucleus, these metabolites directly modulate chromatin-modifying enzymes and regulators of genomic integrity. Acetyl-CoA and related acyl-CoAs fuel histone acyltransferases, facilitating gene activation. NAD⁺ mediates sirtuin-dependent deacetylation. ATP powers chromatin remodelling and histone phosphorylation, while dNTPs sustain DNA replication and damage repair. SAM donates its methyl group for DNA and histone methyltransferases, generating S-adenosylhomocysteine (SAH). FAD functions as a cofactor for lysine-specific demethylase 1 (LSD1). α-KG promotes the activity of TET DNA demethylases and Jumonji-domain histone demethylases, whereas 2HG, succinate and fumarate inhibit these enzymes, causing hypermethylation. Together, these processes align epigenetic regulation with mitochondrial metabolic flux and cellular energetic requirements. Created in BioRender. Das C (2026) https://BioRender.com/ms099px.

A paradigm shift occurred in the cellular stress response pathways with intricate metabolic-epigenetic crosstalk, forming the crux of mito-nuclear collaboration (Fig. 2A, B). Upon sensing stresses like electron transport chain defect, oxidative injury, proteotoxic burden, etc., mitochondria alter their metabolic yield, directly affecting the nuclear epigenome (Chen et al, 2026; Santos, 2021; Quirós et al, 2016). A plethora of mitochondrial metabolites like acetyl-CoA, α-ketoglutarate, succinate, NAD⁺, etc., are important cofactors, modulators or substrates for several enzymes modifying the chromatin landscape. Thus, changes in the metabolite pools have immediate consequences for the gene expression patterns as a result of altered histone acetylation and methylation profiles (Sahu and Lu, 2025; Su et al, 2016). By modulating the availability of metabolites that serve as substrates, cofactors or inhibitors of chromatin-modifying enzymes, mitochondria translate transient stress-induced metabolic changes into alterations in histone and DNA modification patterns. This aids in priming the organism for future stress responses, influencing cell fate decisions and essentially connecting organellar crosstalk to sustained transcriptional signature.

### One-carbon metabolism-mediated chromatin methylation

Epigenetic regulation of gene transcription and the formation of euchromatin and heterochromatin are fundamentally governed by DNA and histone methylation (Fig. 2A,B). These modifications are dynamically connected with one-carbon (1C) metabolism. Mitochondria serve as a primary source of 1C units that are derived from serine catabolism via Serine hydroxymethyltransferase 2 (SHMT2), a mitochondrial enzyme. These 1C units are exported to the cytosol as formate to fuel the methionine cycle and S-adenosylmethionine (SAM) production (Ducker and Rabinowitz, 2017). SAM is the universal methyl group donor for DNA, RNA and histone methylation, thus regulating gene expression. However, the methylation potential of the cell is not determined solely by the abundance of SAM. The balance between SAM and its immediate inhibitory byproduct, S-adenosylhomocysteine (SAH), is a crucial determinant for methylation to occur (Mentch et al, 2015). This SAM:SAH ratio is rapidly altered by changes in the availability of methionine, serine, threonine or folate (Ducker and Rabinowitz, 2017; Shyh-Chang et al, 2013). The altered ratio, in turn, modulates the activity of methyltransferases, reshaping the H3K4me3 and H3K9me3 landscape (Petrova et al, 2023; Ulanovskaya et al, 2013). Most importantly, distinct methyltransferases have different affinities for SAM. This results in some methylation marks changing more readily than others (Mentch and Locasale, 2016). Moreover, the 1C cycle is directly driven by Vitamin B12, an essential cofactor for methionine synthase. Its deficiency results in reduced production of methionine and SAM, decreasing the availability of this methyl group donor (Laranjeira et al, 2024).

Thus, by regulating crucial steps of the folate-methionine cycle, mitochondria impact the methylation pattern of the epigenome and its transcriptional outcome.

### TCA cycle intermediates governing gene transcription

The mitochondria-governed metabolic-epigenetic coupling facilitated by the TCA cycle is a testimony to the fact that this pathway is not merely an energy factory (Fig. 2A). Inside the mitochondria, the TCA cycle maintains nutrient and redox balance. Additionally, several TCA cycle intermediates help control enzymes involved in chromatin modification.

This diversity of TCA cycle-derived metabolites and cofactors involved in epigenetic regulation raises an important question: how are these molecules supplied to the nucleus, and what mechanisms govern their turnover after the utilisation? A conventional explanation would suggest that these metabolites originate exclusively from the mitochondrial TCA cycle and are subsequently transported to the nucleus. However, this model relies on two major assumptions. First, the existence of a direct communication or sensing mechanism between mitochondria and the nucleus, and second, the presence of efficient transport systems capable of delivering these metabolites to their nuclear targets. While no definitive evidence supports a dedicated mitochondria-nuclear sensing system, transport-based delivery of metabolic intermediates to the nucleus would likely be energetically expensive. Given that epigenetic regulation is highly dynamic and tightly controlled, cells must possess mechanisms that allow rapid and precisely regulated supply and removal of metabolites within the nucleus itself. This challenge becomes even more significant as cells must continuously adapt to environmental fluctuations such as nutrient availability and metabolic stress. These external cues influence essential cellular processes, including differentiation, proliferation and stress adaptation, often by rapidly remodelling chromatin structure and transcriptional programs. Such responses require a rapid and precise supply of metabolic cofactors. Beyond the canonical point of view, emerging evidence suggests that cells have evolved a more integrated metabolic architecture involving non-canonical and nuclear TCA cycle pathways (Li et al, 2022; Arnold et al, 2022). These interconnected systems function to bridge mitochondrial metabolism with nuclear chromatin regulation, ensuring localised and timely generation or clearance of epigenetically relevant metabolites.

For example, acetyl-CoA, a central metabolite of several interconnected metabolic pathways, serves as a key substrate for histone acetylation. It can be produced within the mitochondria from pyruvate, fatty acids, β-oxidation, certain amino acids or acetate. Mitochondrial citrate synthase subsequently condenses acetyl-CoA with oxaloacetate to form citrate. This citrate can be exported to the cytosol by the Solute Carrier Family 25 Member 1 (SLC25A1) transporter and is further translocated into the nucleus, where nuclear ATP-citrate lyase (ACLY) reconverts it into acetyl-CoA and oxaloacetate (Wellen et al, 2009). In addition, nuclear Acyl-CoA Synthetase Short-Chain Family Member 2 (ACSS2) and Pyruvate Dehydrogenase (PDH) further enrich the nuclear acetyl-CoA pool by utilising acetate and pyruvate as donors, respectively (Pietrocola et al, 2015; Wiese and Bannister, 2020; Sutendra et al, 2014). Thus, the presence of TCA cycle enzymes inside the nucleus can feed epigenetic regulators and influence chromatin architecture. Further, the acetyl-CoA to CoA ratio links metabolism to chromatin structure and gene expression, as HATs are inhibited by their product CoA (Sivanand et al, 2018). This ratio, rather than acetyl-CoA itself, is a direct reflection of the cellular nutrient status. For example, in the fed state, a high acetyl-CoA/CoA ratio in the nucleus promotes histone acetylation and open chromatin, supporting gene expression. Conversely, in the fasted state, lower acetyl-CoA favours histone deacetylation, causing chromatin compaction (Shi and Tu, 2015).

Beyond histone acetylation, several other epigenetic modifications, including histone succinylation, histone methylation and DNA methylation, also depend on TCA cycle-derived metabolites. For example, members of the amine oxidase family of histone demethylases, such as LSD1, require FAD⁺ as a cofactor to mediate proton transfer during demethylation (Shi et al, 2004). In contrast, Jumonji-domain-containing histone demethylases depend on α-ketoglutarate (α-KG) as a co-substrate, converting α-KG to succinate during catalysis (Tsukada et al, 2006). A similar requirement exists in DNA demethylation, where TET dioxygenases utilise α-KG to oxidise 5-methylcytosine (5mC) sequentially into 5-hydroxymethylcytosine (5hmC), 5-formylcytosine (5fC) and 5-carboxylcytosine (5caC) (Wu and Zhang, 2017). More than sixty α-KG-dependent dioxygenases have been identified in mammalian cells, the majority of which function within the nucleus (Rose et al, 2011). The functioning of these α-KG-dependent dioxygenases depends largely on the balance between α-KG and competitive inhibitory metabolites like succinate and fumarate from the TCA cycle or 2-hydroxyglutarate (2HG), an α-KG-derived metabolite (Baksh and Finley, 2021; Xiao et al, 2012; Sciacovelli et al, 2016; Martínez-Reyes and Chandel, 2020). The presence of essential TCA cycle enzymes, like citrate synthase (CS), aconitase 2 (ACO2), isocitrate dehydrogenase 3 (IDH3) and oxoglutarate dehydrogenase (OGDH), within the nucleus maintains the production of α-KG, succinate and fumarate from the nuclear citrate pool (Nagaraj et al, 2017; Wang et al, 2017). Thus, metabolite ratios such as acetyl-CoA/CoA and α-KG/succinate or fumarate can impact the chromatin state and transcriptional outcome.

Taken together, the mitochondrial metabolite-mediated regulation of chromatin architecture is a well-established phenomenon. The fluctuations in the abundance of such metabolites are translated into locus-specific chromatin changes, rather than bringing about genome-wide alterations. These precise transcriptional programs are modulated by various epigenetic regulatory proteins (Cao et al, 2025; Mondal et al, 2026). However, the exact mechanism by which these epigenetic regulators respond to the metabolic state and exert their functions remains an active area of investigation.

### Fatty acid metabolism mediates histone modification

Apart from the above-mentioned traditional chromatin modifications, emerging studies describe the role of several new histone acylation modifications and their role in transcriptional regulation (Fig. 2B). Beyond their conventional role as energy sources, free fatty acids (FFAs) contribute substantially to cellular pools of acetyl-CoA, propionyl-CoA, butyryl-CoA, crotonyl-CoA, succinyl-CoA and other acyl-CoA intermediates. Fluctuations in the abundance of these metabolites provide a direct mechanism through which cellular metabolic status can influence chromatin structure and transcriptional activity.

Among the various modifications named above, histone acetylation by acetyl-CoA from the cellular acetate pool is most widely characterised. However, other fatty acid-derived metabolites can also facilitate a range of histone acylations, like propionylation, butyrylation, crotonylation and succinylation (Nshanian et al, 2025; Li et al, 2016; Papsdorf and Brunet, 2019). Short-chain fatty acids, particularly butyrate and propionate, are vital mediators of metabolic-epigenetic communication. These metabolites influence gene regulation by serving as precursors for histone acyl modifications, namely butyrylation and propionylation, while also modulating histone deacetylase activity. For instance, butyrate acts as an inhibitor of histone deacetylases (HDACs), increasing histone acetylation and enhancing butyrylation-driven gene activation (Donohoe et al, 2012). Succinylation of histone lysine residues, driven by succinyl-CoA, represents another fatty acid metabolism-linked modification of potential functional importance. It is enriched at transcriptional start sites and active genomic regions, where its presence correlates with increased gene expression, suggesting a role in promoting transcriptional activity (Li et al, 2023; Liu et al, 2021).

Several histone acetyltransferases (HATs) have recently been identified to transfer a variety of acyl groups to lysine residues on histones. For example, p300 promotes crotonylation, butyrylation, glutarylation and succinylation, albeit it is typically identified for acetylation (Goudarzi et al, 2016; Sabari et al, 2015; Kaczmarska et al, 2017). Surprisingly, relative to its acetyltransferase activity, the crotonyltransferase role of p300 exerts a much more potent stimulatory effect on transcription, enhancing gene expression (Sabari et al, 2015). Similarly, histone propionylation can be catalysed by the HATs Gcn5 and p300-CBP-associated factor (PCAF) (Berndsen et al, 2007; Leemhuis et al, 2008).

The dynamics of the histone acylation marks is further regulated by enzymes that remove these modifications. For example, in addition to their well-established deacetylase function, the NAD^+^-dependent sirtuins, SIRT1, SIRT2 and SIRT3 have been identified as histone decrotonylases (Bao et al, 2014). Several other modifications like malonylation, 2-hydroxyisobutyrylation and β-hydroxybutyrylation further expand the repertoire of histone marks that respond to changes in fatty acid metabolism. Acylation of histones can also occur by non-enzymatic covalent modifications (NECMs), bypassing the conventional epigenetic “writer” proteins. The elevated intracellular concentration and intrinsic chemical reactivity of the acyl-CoA metabolites promote their binding to histone lysine residues spontaneously (Maksimovic and David, 2021; Diehl and Muir, 2020). Thus, alterations in fatty acid metabolism can influence chromatin states through both enzymatic and non-enzymatic mechanisms. Together, these emerging histone acyl modifications directly link fatty acid metabolism to transcriptional regulation. While the enzymatic machineries responsible for catalysing the addition and removal of the various histone acylation marks have been characterised, their specific biological roles and regulatory significance are still being elucidated.

Thus, mitochondrial metabolic intermediates, with the help of epigenetic regulators, can regulate the chromatin architecture and gene transcription pattern in a spatiotemporal manner. Similarly, the nuclear genome regulates the availability and abundance of epigenetically relevant mitochondrial metabolite pools by transcribing the crucial enzymes responsible for their production. Therefore, rather than being straightforward linear cause-and-effect mechanisms, the anterograde and retrograde pathways form complementary, interdependent entities to maintain cellular integrity and homeostasis. Further, the compartmentalised mitochondria-derived cellular metabolic pools are not restrained within the boundaries of the cell. During several physiological assaults, these metabolites can be secreted from cells to the extracellular milieu, potentially acting as signalling molecules or biomarkers for specific pathophysiological states.

## Circulating metabolites: regulating systemic interactions

**Figure 3:**
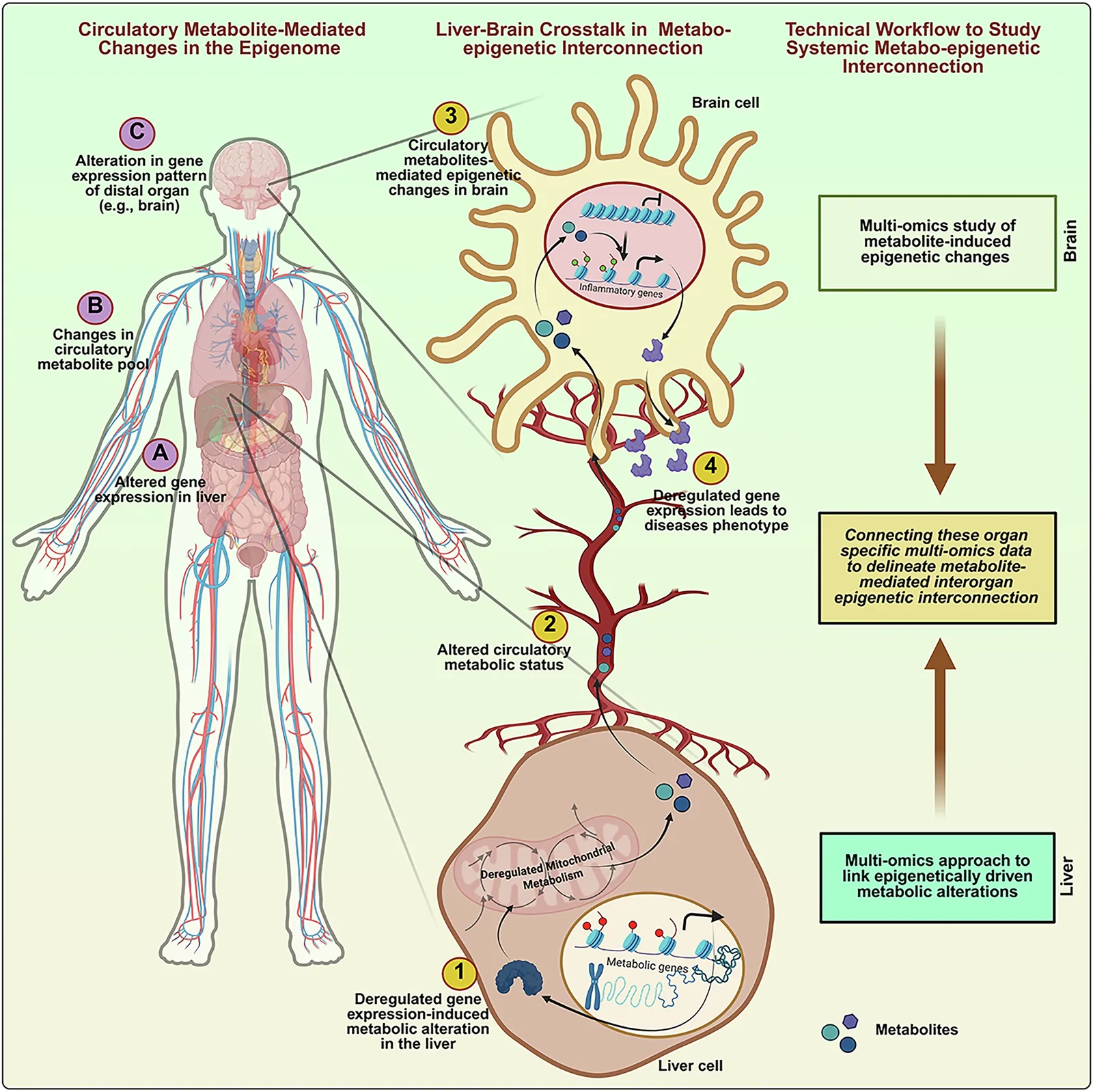
Proposed model of metabolite-mediated interorgan crosstalk and its potential contribution to epigenetic and metabolic reprogramming across the liver–brain axis. Alterations in hepatic gene expression, which may occur under different dietary or metabolic conditions, can reshape the hepatic metabolic landscape and potentially alter the pool of circulating metabolites. These metabolic intermediates may reach distal organs like the brain, where they could cause epigenetic rewiring and changes in gene expression patterns, thereby contributing to disease-associated phenotypes. The left panels (**A**–**C**) illustrate putative systemic effects, while the middle panel (1–4) depicts proposed intercellular mechanisms. The epigenetic modifications depicted may regulate gene expression, but are yet to be explored in the context of metabolite-mediated interorgan crosstalk. The right panel highlights proposed multi-omics approaches that may facilitate the mapping of organ-specific epimetabolic states and help evaluate these interorgan communication pathways. Created in BioRender. Das C (2026) https://BioRender.com/l3x7x4r.

Unicellular organisms manage all their life processes within a tiny cell, handling their internal milieu locally. In contrast, complex multicellular life forms survive through the seamless coordinated functioning of their organs. Such interorgan crosstalk forms the fundamental network to maintain systemic homeostasis. Conventionally, long-distance communication within the body has been predominantly attributed to hormones and neuronal pathways. In addition, the brain has been typically positioned as the endocrine signalling nexus to distant organs. However, recent studies highlight a more reciprocal communication where peripheral organs actively secrete metabolites as circulating signalling molecules, relaying information back to the central nervous system (Fig. 3). For instance, hepatic overexpression of ACOT12 increases blood acetate levels, leading to the accumulation of acetyl-CoA in the ventral hippocampus of the brain. This results in histone acetylation-mediated activation of anti-inflammatory genes, suppressing neuroinflammation (Cao et al, 2025). Similarly, consumption of a high-fat diet (HFD) increases serum triglyceride and glucose levels, leading to reduced ACLY and ACSS2 expression in liver and adipose tissues, which ultimately lowers the acetyl-CoA pool in these cells. This reduction limits histone acetylation in a tissue-specific manner. For example, H3K23ac and H3K18ac are decreased in adipose tissues and the pancreas, respectively (Carrer et al, 2017). Furthermore, global partial inhibition of MCT1 (a transporter of lactate, ketone bodies and short-chain fatty acids) reduces diet-induced hepatic steatosis and preserves brain function (Hadjihambi et al, 2023). This suggests that systemic metabolite-mediated crosstalk with the neurological system can be modulated to shape metabolic responses across tissues. Likewise, mtDNA-encoded signals, such as N-formyl peptides, link organellar abnormalities to disease phenotypes like neurodegeneration (Zhang et al, 2010). In response to injured mitochondria, these short amino acid chains function as damage-associated molecular patterns (DAMPs) and are released into the cytosol or extracellular space. Upon entering circulation, these peptides interact with immune receptors, subsequently triggering ERK1/2 and p38 MAPK pathway activation and promoting inflammatory responses (Hazeldine et al, 2015; Kwon et al, 2021). Systemic metabolite signalling is an emerging field, and much remains to be discovered. The mechanisms underlying how metabolites signal between tissues and how they can be targeted in disease are still poorly understood.

Given that circulating metabolites can serve as mediators of interorgan communication, perturbations in this metabolic signalling cascade can have pathological consequences. The capacity of the circulating metabolites to bridge distant organ systems underscores a deeper, intrinsic dependency of the cellular machinery on metabolic fluctuating states. As described previously, the metabolites connect mitochondrial function with the epigenome, shaping gene expression patterns. This local, bidirectional communication, when disrupted, may result in disease phenotypes. This may be the effect of metabolic dysfunction, altered metabolite levels or mutations in epigenetic regulators. Studying these intracellular and intercellular networks uncovers the molecular basis of “epimetabopathies,” a group of disorders where defects in epigenetic–metabolic crosstalk serve as the primary driver of several ailments.

## “Epimetabopathies”: epigenetic–metabolic dysfunction as drivers of pathophysiology

The interconnected network of nuclei-mitochondrial crosstalk plays an important role in cellular homeostasis and physiological pathogenesis. Any disruption in this bidirectional signalling can alter the nuclear epigenome, hampering mitochondrial metabolism. Conversely, deregulated mitochondrial metabolism can perturb chromatin architecture, resulting in complex disease pathology. For example, abnormalities in the epigenetic reader protein Methyl-CpG-binding protein 2 (MeCP2) can amend the gene expression pattern of mitochondrial metabolic enzymes, leading to neurological disorders like Rett Syndrome (RTT) (Gold et al, 2024; Buchovecky et al, 2013) and MECP2 Duplication Syndrome (MDS) (Yang et al, 2024; Rizvi et al, 2024). In normal physiological conditions, MeCP2 recognises methylated CpG regions of the genome and promotes the recruitment of Histone Deacetylase 3 (HDAC3) and NCOR-SMRT complex to repress certain genes (Lewis et al, 1992; Lyst et al, 2013; Lagger et al, 2017). Similarly, it can also recognise G-quadruplex structure in the gene promoter and help recruit the CREB1 co-activation complex and RNA polymerase II to initiate gene transcription (Gold et al, 2024; Chahrour et al, 2008). In Rett Syndrome (RTT), a loss-of-function mutation in the MECP2 gene increases transcription of the Uqcrc1 gene, which encodes a subunit of Complex III of the mitochondrial electron transport chain, leading to heightened ROS production in the brain (Kriaucionis et al, 2006; Chandel et al, 2000). Similarly, in the absence of functional MeCP2, the mitochondrial COX1 gene gets repressed in the frontal cortex of RTT patients, leading to reduced mitochondrial Complex IV activity (Gibson et al, 2010). On the other hand, duplication of the MECP2 gene acts as a dosage-sensitive switch, altering the metabolic landscape. This results in upregulation of nucleotide biosynthesis and ROS production in the brain and excess testosterone synthesis in the testis, leading to the pathological condition of MDS (D’Mello, 2021). Excess activity of MeCP2 disrupts the electron flow in the mitochondria due to the decompaction of Complex III of ETC, resulting in redox imbalance and reduced oxygen consumption, causing hypoxia in the central nervous system (CNS) (Signorini et al, 2016). MeCP2-mediated inhibition of Complex III promotes the production of aspartate and the TCA cycle intermediate, fumarate, in the mitochondria. This assists in the formation of purinosomes in the cytosol. Further, the formation of purinosomes elevates the level of the cellular nucleotide and xanthine pool, causing double-strand breaks in the nuclei of glial cells (Cappuccio et al, 2025). Beyond the CNS, MeCP2 also exercises significant regulatory roles in the testis. It upregulates the transcription of the luteinizing hormone receptor (LHCGR) by employing the CREB1 co-activator complex. Simultaneously, MeCP2 binds to the methylated CpG islands in the ROR-α locus, suppressing its expression. These mechanisms together increase androgen production, resulting in the development of secondary sexual characteristics in MDS patients (Wang et al, 2021).

Similar to epigenome-mediated regulation of metabolism, alterations in the metabolic pool can also change the epigenetic landscape, rearranging the gene expression machinery, which may cause severe physiological imbalance. For example, studies of famine-exposed individuals revealed a reduced DNA methylation status at IGF2 gene loci, leading to obesity and dyslipidaemia (Kaelin and McKnight, 2013). Similarly, an alcohol-mediated nuclear translocation of ACSS2 enriches the nuclear acetyl-CoA pool. This promotes PCAF-mediated histone H3K9 acetylation of FASN and ACACA gene promoters. Upon activation, FASN and ACACA promote de novo lipogenesis, leading to hepatic steatosis (Lu et al, 2023).

Alongside physiological abnormalities, alterations in cellular and systemic metabolic pools, by dietary interventions or altered gene activation, can also protect against tissue-specific pathophysiological states. For example, extracellular metabolites like α-ketoglutarate (α-KG) can bind to the purinergic receptor, P2RX4 and promote the expression of SLC25A11 in the mitochondrial membrane, leading to the enrichment of the cytosolic α-KG pool. Increase in cytosolic α-KG induces its diffusion into the nucleus and acts as a cofactor for histone demethylases and activates seprina1e (serine or cysteine peptidase inhibitor, clade A, member 1e) gene transcription by facilitating histone H3K27me3 demethylation at its promoter, thus suppressing gluconeogenesis and hyperglycaemia (Yuan et al, 2022). Further, a ketone body, d-β-hydroxybutyrate (βOHB), enriched diet or caloric restriction increases the cellular and plasma abundance of βOHB. Heightened βOHB promotes histone acetylation at FOXO3A and MT2 gene promoters by suppressing the activity of HDACs, leading to protection against oxidative stress (Shimazu et al, 2013).

While substantial evidence supports that disrupting the epigenetic–metabolic axis can drive disease pathogenesis and, when appropriately modulated, offer therapeutic benefits, this field remains in its infancy. So far, only a handful of metabolite-epigenome crosstalk pathways and their molecular mechanisms have been clearly mapped. Tracking the transient metabolite fluxes and studying their interactions with spatiotemporal tissue-specific precision remains challenging. As advanced metabolomic, epigenomic and spatial multi-omics profiling tools develop, a more comprehensive overview of epimetabopathies is expected to emerge.

## Next-generation diagnostics and therapeutics for epimetabopathies

Recent advancements in high-resolution techniques like organelle-resolved metabolomics, single-cell multi-omics and spatial epigenomics have revolutionised our understanding of disease pathogenesis. Mass spectrometry imaging (MSI), one of the most powerful tools for MS-based single-cell metabolomics, uniquely combines molecular specificity with spatial resolution. It helps identify the localisation of different metabolites within tissues, making it easier to comprehend how cells interact with their surroundings. The spatial information thus obtained links metabolic changes to local epigenetic dysfunction, aiding identification of region-specific disease alterations (Taylor et al, 2021). Complementing this approach, single-cell multi-omics enables the simultaneous measurement of genomic, epigenomic, transcriptomic, proteomic and metabolomic data within individual cells (Kumar et al, 2024). Further, spatially resolved multi-omics identify metabolic changes across various cell types (Sun et al, 2023). Together, these tools pave the way for a deeper, multilayered understanding of epimetabopathies.

Translating the mechanistic understanding to effective therapies demands context-specific strategies. For example, CRISPR/Cas9 editing offers therapeutic potential for directly reprogramming metabolic pathways to correct genetic metabolic disorders (Pankowicz et al, 2016; Rutten et al, 2019). Epigenetic drugs, like inhibitors of DNA methyltransferases or histone deacetylases, can reverse disease-causing abnormal chromatin states and restore healthy cellular function (Heerboth et al, 2014). Other examples include metabolite mimetics such as 2-hydroxyglutarate that can alter the epigenetic–metabolic axis by inhibiting α-ketoglutarate-dependent dioxygenases, thereby modifying gene expression patterns (Xu et al, 2011). Several mitochondrial modulators, namely, metformin, resveratrol, MitoQ, etc., can also be used to restore mitochondrial function by enhancing energy production and biogenesis, reducing oxidative stress and correcting the underlying metabolic imbalance (Mei et al, 2025; Kim et al, 2024).

Although targeting the metabolic-epigenetic axis holds a lot of therapeutic potential, several challenges are yet to be addressed (Box 1). Therapeutic techniques often lack tissue specificity, causing unintended side effects in non-targeted pathways. Metabolic heterogeneity and dynamic compartmentalisation of metabolites further complicate systemic interventions. Thus, altering key metabolic nodes can have divergent outcomes in different organs, highlighting the need for cell-type or tissue-specific strategies.

Box 1 In need of answers

1. Do different mitochondrial subpopulations within a single cell send distinct signals to influence the chromatin state?
2. Can mitochondrial metabolites regulate the formation of liquid-liquid phase-separated condensates within the nucleus?
3. Which circulating metabolites function as systemic epigenetic regulators?
4. Can circulating metabolites serve as stable biomarkers for detecting and staging epimetabopathies?
5. Can a circulating metabolite associated with a particular disease indicate the secondary organ damage resulting from the primary condition?
6. Are epimetabopathies reversible through metabolic interventions, or does the epigenetic memory persist despite restored mitochondrial function and prevent recovery?
7. Is it possible to achieve cell-type-specific epigenetic editing via metabolic interventions?

## Conclusions and perspectives

Long before complex organisms like animals or plants had taken shape, an ancient free-living bacterium slipped inside a host cell, just like a “Trojan Horse”. However, rather than an invasion, it was an epic symbiotic event that gave birth to mitochondria. While the proteobacteria provided the host with ATP via oxidative phosphorylation, the host gave protection and nutrients. Instead of digesting the invader, the host tolerated it, as it gained a competitive advantage in the oxygen-rich environment. Similarly, cyanobacteria entered different cells and gave rise to today’s chloroplasts in plants. The bacterial endosymbiosis that made the mitochondria probably occurred earlier in history, as all modern-day eukaryotes, irrespective of plant or animal lineage, possess them. In addition, the mitochondria adapt to tissue-specific requirements in an organism. In humans, for example, they are highly oxidative and specialised for continuous ATP production in the heart. Liver mitochondria are metabolically more versatile, while in brown fat, the adipose mitochondria are involved in thermogenesis via uncoupled respiration.

Mitochondria possess their own circular DNA (mtDNA), 55S mitoribosomes and self-replicating potential, albeit their activity and biogenesis are regulated by the nucleus. Over the course of evolution, the nucleus thumped its autonomy via anterograde control over the mitochondria. Retrograde signalling, conversely, helped these organelles to whisper their structural integrity and functional state back to the nucleus. This crosstalk happens to be life’s symphony, and at the heart of this partnership lie the metabolites. These chemical messengers help maintain physiological balance. They synchronise mtDNA replication with cell cycle progression, coordinate energy requirements with cellular demands and maintain overall quality control. Further, metabolites like SAM, acetyl-CoA, α-ketoglutarate, succinate, NAD⁺ and ATP moonlight as epigenetic regulators, tweaking gene expression and integrity.

When this intricate mito-nuclear communication derails, it can give rise to serious diseases that we have termed “epimetabopathies”. Our perspective is that, rather than a single pathway defect, these ailments occur due to metabolic-epigenetic crosstalk going haywire. It would be interesting to study if certain epigenetic modifications influence mitochondrial architectural and functional state in such a manner that predisposes an individual to metabolic dysfunction, even before the onset of a diagnosable disease. Conversely, as several metabolic pathways can set the stage for chromatin modifications, it is essential to know the precise spatiotemporal dynamics of these metabolic signals. Do disruptions in these metabolic checkpoints alter the epigenome, committing cells into a diseased state? Future studies integrating single-cell multi-omics with mitochondrial activity and chromatin state will be an interesting avenue to pursue. Organellar metabolomics can further be combined with spatial epigenomics to investigate potential therapeutic targets.

As metabolites are the main mediators of an efficient mito-nuclear signalling, bold therapeutic ideas on metabolite-based interventions are gradually emerging. Examples include NAD^+^ boosters, ketogenic diets to increase fatty acid oxidation, calorie restriction and intermittent fasting to increase mitochondrial biogenesis and reduce ROS generation. Although it is clear that food and small molecules can restore cellular function, the potential side effects of such metabolic therapies are not yet well understood. Such interventions ripple through dynamic interconnected signalling pathways within the cell. Boosting one network might have dire consequences on the other, overstimulating it, altering redox balance or causing any other unforeseen catastrophe. Flooding the cells with exogenous metabolites could have serious long-term consequences, manipulating the intricate organellar crosstalk. It is essential to address these topics and questions in order to target the metabolic-epigenetic pathways for precise therapeutic intervention in complex diseases.

## Supplementary information

Peer Review File

## Glossary

α-KG: Alpha-Ketoglutarate
ATP: Adenosine Triphosphate
CNS: Central Nervous System
CoA: Coenzyme A
DDR: DNA Damage Response
dGTP: Deoxyguanosine Triphosphate
DHODH: Dihydroorotate Dehydrogenase
dNTP: Deoxynucleotide Triphosphate
ETC: Electron Transport Chain
FGF21: Fibroblast Growth Factor 21
HAT: Histone Acetyltransferase
HDAC: Histone Deacetylase
HFD: High-Fat Diet
LSD1: Lysine-Specific Demethylase 1
MCT1: Monocarboxylate Transporter 1
MDS: MECP2 Duplication Syndrome
MeCP2: Methyl-CpG-Binding Protein 2
mtDNA: Mitochondrial DNA
mitoROS: Mitochondrial Reactive Oxygen Species
NAD⁺: Nicotinamide Adenine Dinucleotide
nDNA: Nuclear DNA
NF-κB: Nuclear Factor Kappa B
NRF-1: Nuclear Respiratory Factor 1
NRF-2: Nuclear Respiratory Factor 2
OXPHOS: Oxidative Phosphorylation
PARP: Poly (ADP-ribose) Polymerase
PCAF: p300/CBP-Associated Factor
PGC-1α: Peroxisome Proliferator-Activated Receptor Gamma Co-activator 1 Alpha
PPAR: Peroxisome Proliferator-Activated Receptor
ROS: Reactive Oxygen Species
RTT: Rett Syndrome
SAM: S-Adenosylmethionine
SIRT1: Sirtuin 1
TCA: Tricarboxylic Acid
TET: Ten-Eleven Translocation
TFAM: Mitochondrial Transcription Factor A
UPRmt: Mitochondrial Unfolded Protein Response
XPA: Xeroderma Pigmentosum Group A
YY1: Yin Yang 1

## References

[CR1] Al-Mehdi AB, Pastukh VM, Swiger BM, Reed DJ, Patel MR, Bardwell GC, Pastukh VV, Alexeyev MF, Gillespie MN (2012) Perinuclear mitochondrial clustering creates an oxidant-rich nuclear domain required for hypoxia-induced transcription. Sci Signal 5:ra47 10.1126/scisignal.2002712 PMC3565837 22763339

[CR2] Arnold PK, Jackson BT, Paras KI, Brunner JS, Hart ML, Newsom OJ, Alibeckoff SP, Endress J, Drill E, Sullivan LB et al (2022) A non-canonical tricarboxylic acid cycle underlies cellular identity. Nature 603:477–481 35264789 10.1038/s41586-022-04475-w PMC8934290

[CR3] Awoyomi OF, Gorospe CM, Das B, Mishra P, Sharma S, Diachenko O, Nilsson AK, Tran P, Wanrooij PH, Chabes A (2025) RRM2B deficiency causes dATP and dGTP depletion through enhanced degradation and slower synthesis. Proc Natl Acad Sci USA 122:e2503531122 10.1073/pnas.2503531122 PMC12037051 40244665

[CR4] Bai P, Cantó C (2012) The role of PARP-1 and PARP-2 enzymes in metabolic regulation and disease. Cell Metab 16:290–295 22921416 10.1016/j.cmet.2012.06.016

[CR5] Bai P, Cantó C, Oudart H, Brunyánszki A, Cen Y, Thomas C, Yamamoto H, Huber A, Kiss B, Houtkooper RH et al (2011) PARP-1 inhibition increases mitochondrial metabolism through SIRT1 activation. Cell Metab 13:461–468 21459330 10.1016/j.cmet.2011.03.004 PMC3086520

[CR6] Baker SA, Rutter J (2023) Metabolites as signalling molecules. Nat Rev Mol Cell Biol 24:355–374 36635456 10.1038/s41580-022-00572-w

[CR7] Baksh SC, Finley LWS (2021) Metabolic coordination of cell fate by α-ketoglutarate-dependent dioxygenases. Trends Cell Biol 31:24–36 33092942 10.1016/j.tcb.2020.09.010 PMC7748998

[CR8] Bao X, Wang Y, Li X, Li XM, Liu Z, Yang T, Wong CF, Zhang J, Hao Q, Li XD (2014) Identification of ‘erasers’ for lysine crotonylated histone marks using a chemical proteomics approach. eLife 3:e02999 10.7554/eLife.02999 PMC4358366 25369635

[CR9] Bell EL, Klimova TA, Eisenbart J, Schumacker PT, Chandel NS (2007) Mitochondrial reactive oxygen species trigger hypoxia-inducible factor-dependent extension of the replicative life span during hypoxia. Mol Cell Biol 27:5737–5745 17562866 10.1128/MCB.02265-06 PMC1952129

[CR10] Berndsen CE, Albaugh BN, Tan S, Denu JM (2007) Catalytic mechanism of a MYST family histone acetyltransferase. Biochemistry 46:623 17223684 10.1021/bi602513x PMC2752042

[CR11] Birsoy K, Wang T, Chen WW, Freinkman E, Abu-Remaileh M, Sabatini DM (2015) An essential role of the mitochondrial electron transport chain in cell proliferation is to enable aspartate synthesis. Cell 162:540–551 26232224 10.1016/j.cell.2015.07.016 PMC4522279

[CR12] Biswas G, Adebanjo OA, Freedman BD, Anandatheerthavarada HK, Vijayasarathy C, Zaidi M, Kotlikoff M, Avadhani NG (1999) Retrograde Ca2+ signaling in C2C12 skeletal myocytes in response to mitochondrial genetic and metabolic stress: a novel mode of inter-organelle crosstalk. EMBO J 18:522–533 9927412 10.1093/emboj/18.3.522 PMC1171145

[CR13] Biswas G, Anandatheerthavarada HK, Zaidi M, Avadhani NG (2003) Mitochondria to nucleus stress signaling: a distinctive mechanism of NFkappaB/Rel activation through calcineurin-mediated inactivation of IkappaBbeta. J Cell Biol 161:507–519 12732617 10.1083/jcb.200211104 PMC2172940

[CR14] Blättler SM, Verdeguer F, Liesa M, Cunningham JT, Vogel RO, Chim H, Liu H, Romanino K, Shirihai OS, Vazquez F et al (2012) Defective mitochondrial morphology and bioenergetic function in mice lacking the transcription factor Yin Yang 1 in skeletal muscle. Mol Cell Biol 32:3333–3346 22711985 10.1128/MCB.00337-12 PMC3434543

[CR15] Buchovecky CM, Turley SD, Brown HM, Kyle SM, McDonald JG, Liu B, Pieper AA, Huang W, Katz DM, Russell DW et al (2013) A suppressor screen in Mecp2 mutant mice implicates cholesterol metabolism in Rett syndrome. Nat Genet 45:1013–1020 23892605 10.1038/ng.2714 PMC3837522

[CR16] Butow RA, Avadhani NG (2004) Mitochondrial signaling: the retrograde response. Mol Cell 14:1–15 15068799 10.1016/s1097-2765(04)00179-0

[CR17] Cantó C, Menzies KJ, Auwerx J (2015) NAD+ metabolism and the control of energy homeostasis: a balancing act between mitochondria and the nucleus. Cell Metab 22:31–53 26118927 10.1016/j.cmet.2015.05.023 PMC4487780

[CR18] Cao K, Riley JS, Heilig R, Montes-Gómez AE, Vringer E, Berthenet K, Cloix C, Elmasry Y, Spiller DG, Ichim G et al (2022) Mitochondrial dynamics regulate genome stability via control of caspase-dependent DNA damage. Dev Cell 57:1211–1225.e6 35447090 10.1016/j.devcel.2022.03.019 PMC9616799

[CR19] Cao Y, Tang L, Du K, Paraiso K, Sun Q, Liu Z, Ye X, Fang Y, Yuan F, Chen H et al (2021) Anterograde regulation of mitochondrial genes and FGF21 signaling by hepatic LSD1. JCI Insight 6:e147692 10.1172/jci.insight.147692 PMC8492328 34314389

[CR20] Cao Y, Zhao Y, Deng T, Zhou Q, Hu G, Hu ZL, Jiang YY, Yang XH, Wang F, Wu PF et al (2025) Hepatic acetyl-CoA metabolism modulates neuroinflammation and depression susceptibility via acetate. Cell Metab 37:2185–2201.e8 40992374 10.1016/j.cmet.2025.08.010

[CR21] Cappuccio G, Qi G, Qin X, Khalil SM, Hunyara J, Li Y, Mathews J, Soriano A, Osenberg S, Sing S et al (2025) MECP2 duplication uncouples mitochondrial and purine metabolism during neuronal maturation. Preprint at https://www.biorxiv.org/content/10.64898/2025.12.24.696399v1.full

[CR22] Carrer A, Parris JLD, Trefely S, Henry RA, Montgomery DC, Torres A, Viola JM, Kuo YM, Blair IA, Meier JL et al (2017) Impact of a high-fat diet on tissue acyl-CoA and histone acetylation levels. J Biol Chem 292:3312–3322 28077572 10.1074/jbc.M116.750620 PMC5336165

[CR23] Chahrour M, Sung YJ, Shaw C, Zhou X, Wong STC, Qin J, Zoghbi HY (2008) MeCP2, a key contributor to neurological disease, activates and represses transcription. Science 320:1224–1229 18511691 10.1126/science.1153252 PMC2443785

[CR24] Chandel NS (2015) Evolution of mitochondria as signaling organelles. Cell Metab 22:204–206 26073494 10.1016/j.cmet.2015.05.013

[CR25] Chandel NS, McClintock DS, Feliciano CE, Wood TM, Melendez JA, Rodriguez AM, Schumacker PT (2000) Reactive oxygen species generated at mitochondrial complex III stabilize hypoxia-inducible factor-1alpha during hypoxia: a mechanism of O_2_ sensing. J Biol Chem 275:25130–25138 10833514 10.1074/jbc.M001914200

[CR26] Chau CMA, Evans MJ, Scarpulla RC (1992) Nuclear respiratory factor 1 activation sites in genes encoding the gamma-subunit of ATP synthase, eukaryotic initiation factor 2 alpha, and tyrosine aminotransferase. Specific interaction of purified NRF-1 with multiple target genes. J Biol Chem 267:6999–7006 1348057

[CR27] Chen G, Dong H, Tian Y (2026) Mito-nuclear communication: from cellular responses to organismal health. Mol Cell 86:522–532 41610858 10.1016/j.molcel.2026.01.001

[CR28] Croteau DL, Fang EF, Nilsen H, Bohr VA (2017) NAD+ in DNA repair and mitochondrial maintenance. Cell Cycle 16:491 28145802 10.1080/15384101.2017.1285631 PMC5384578

[CR29] Cunningham JT, Rodgers JT, Arlow DH, Vazquez F, Mootha VK, Puigserver P (2007) mTOR controls mitochondrial oxidative function through a YY1-PGC-1alpha transcriptional complex. Nature 450:736–740 18046414 10.1038/nature06322

[CR30] Desai R, East DA, Hardy L, Faccenda D, Rigon M, Crosby J, Alvarez MS, Singh A, Mainenti M, Hussey LK et al (2020) Mitochondria form contact sites with the nucleus to couple prosurvival retrograde response. Sci Adv 6:eabc9955 10.1126/sciadv.abc9955 PMC11206220 33355129

[CR31] Desler C, Lykke A, Rasmussen LJ (2010) The effect of mitochondrial dysfunction on cytosolic nucleotide metabolism. J Nucleic Acids 2010:701518 20862377 10.4061/2010/701518 PMC2938461

[CR32] Desler C, Munch-Petersen B, Stevnsner T, Matsui SI, Kulawiec M, Singh KK, Rasmussen LJ (2007) Mitochondria as determinant of nucleotide pools and chromosomal stability. Mutat Res - Fundam Mol Mech Mutagen 625:112–124 10.1016/j.mrfmmm.2007.06.002 17658559

[CR33] Diehl KL, Muir TW (2020) Chromatin as a key consumer in the metabolite economy. Nat Chem Biol 16:620–629 32444835 10.1038/s41589-020-0517-x PMC7258299

[CR34] D’Mello SR (2021) MECP2 and the biology of MECP2 duplication syndrome. J Neurochem 159:29–60 33638179 10.1111/jnc.15331

[CR35] Donohoe DR, Collins LB, Wali A, Bigler R, Sun W, Bultman SJ (2012) The Warburg effect dictates the mechanism of butyrate-mediated histone acetylation and cell proliferation. Mol Cell 48:612–626 23063526 10.1016/j.molcel.2012.08.033 PMC3513569

[CR36] Ducker GS, Rabinowitz JD (2017) One-carbon metabolism in health and disease. Cell Metab 25:27–42 27641100 10.1016/j.cmet.2016.08.009 PMC5353360

[CR37] Etchegaray JP, Mostoslavsky R (2016) Interplay between metabolism and epigenetics: a nuclear adaptation to environmental changes. Mol Cell 62:695–711 27259202 10.1016/j.molcel.2016.05.029 PMC4893201

[CR38] Evans DR, Guy HI (2004) Mammalian pyrimidine biosynthesis: fresh insights into an ancient pathway. J Biol Chem 279:33035–33038 15096496 10.1074/jbc.R400007200

[CR39] Fang EF, Scheibye-Knudsen M, Brace LE, Kassahun H, Sengupta T, Nilsen H, Mitchell JR, Croteau DL, Bohr VA (2014) Defective mitophagy in XPA via PARP-1 hyperactivation and NAD +/SIRT1 reduction. Cell 157:882–896 24813611 10.1016/j.cell.2014.03.026 PMC4625837

[CR40] Fang EF, Scheibye-Knudsen M, Chua KF, Mattson MP, Croteau DL, Bohr VA (2016) Nuclear DNA damage signalling to mitochondria in ageing. Nat Rev Mol Cell Biol 17:308–321 26956196 10.1038/nrm.2016.14 PMC5161407

[CR41] Fang JX, Uchiumi T, Yagi M, Matsumoto S, Amamoto R, Takazaki S, Yamaza H, Nonaka K, Kang D (2013) Dihydro-orotate dehydrogenase is physically associated with the respiratory complex and its loss leads to mitochondrial dysfunction. Biosci Rep 33:217–227 10.1042/BSR20120097 PMC3564035 23216091

[CR42] Finck BN, Kelly DP (2006) PGC-1 coactivators: inducible regulators of energy metabolism in health and disease. J Clin Investig 116:615 16511594 10.1172/JCI27794 PMC1386111

[CR43] Formentini L, Sánchez-Aragó M, Sánchez-Cenizo L, Cuezva JMC (2012) The mitochondrial ATPase inhibitory factor 1 triggers a ROS-mediated retrograde prosurvival and proliferative response. Mol Cell 45:731–742 22342343 10.1016/j.molcel.2012.01.008

[CR44] Ghose R, Pezzano F, Badia R, Kourtis S, Sheraj I, Das S, Gañez Zapater A, Ghose U, Musa-Afaneh S, Espinar L et al (2025) Mitochondria-derived nuclear ATP surge protects against confinement-induced proliferation defects. Nat Commun 16:6613 10.1038/s41467-025-61787-x PMC12310956 40738970

[CR45] Gibson JH, Slobedman B, KN H, Williamson SL, Minchenko D, El-Osta A, Stern JL, Christodoulou J (2010) Downstream targets of methyl CpG binding protein 2 and their abnormal expression in the frontal cortex of the human Rett syndrome brain. BMC Neurosci 11:53 20420693 10.1186/1471-2202-11-53 PMC2881102

[CR46] Gold WA, Percy AK, Neul JL, Cobb SR, Pozzo-Miller L, Issar JK, Ben-Zeev B, Vignoli A, Kaufmann WE (2024) Rett syndrome. Nat Rev Dis Prim 10:84 39511247 10.1038/s41572-024-00568-0

[CR47] Goudarzi A, Zhang D, Huang H, Barral S, Kwon OK, Qi S, Tang Z, Buchou T, Vitte AL, He T et al (2016) Dynamic competing histone H4 K5K8 acetylation and butyrylation are hallmarks of highly active gene promoters. Mol Cell 62:169–180 27105113 10.1016/j.molcel.2016.03.014 PMC4850424

[CR48] Guo X, Aviles G, Liu Y, Tian R, Unger BA, Lin YHT, Wiita AP, Xu K, Correia MA, Kampmann M (2020) Mitochondrial stress is relayed to the cytosol by an OMA1-DELE1-HRI pathway. Nature 579:427–432 32132707 10.1038/s41586-020-2078-2 PMC7147832

[CR49] Hadjihambi A, Konstantinou C, Klohs J, Monsorno K, Le Guennec A, Donnelly C, Cox IJ, Kusumbe A, Hosford PS, Soffientini U et al (2023) Partial MCT1 invalidation protects against diet-induced non-alcoholic fatty liver disease and the associated brain dysfunction. J Hepatol 78:180–190 35995127 10.1016/j.jhep.2022.08.008

[CR50] Hämäläinen RH, Landoni JC, Ahlqvist KJ, Goffart S, Ryytty S, Rahman MO, Brilhante V, Icay K, Hautaniemi S, Wang L et al (2019) Defects in mtDNA replication challenge nuclear genome stability through nucleotide depletion and provide a unifying mechanism for mouse progerias. Nat Metab 1:958–965 32694840 10.1038/s42255-019-0120-1

[CR51] Harding HP, Zhang Y, Zeng H, Novoa I, Lu PD, Calfon M, Sadri N, Yun C, Popko B, Paules R et al (2003) An integrated stress response regulates amino acid metabolism and resistance to oxidative stress. Mol Cell 11:619–633 12667446 10.1016/s1097-2765(03)00105-9

[CR52] Hazeldine J, Hampson P, Opoku FA, Foster M, Lord JM (2015) N-Formyl peptides drive mitochondrial damage associated molecular pattern induced neutrophil activation through ERK1/2 and P38 MAP kinase signalling pathways. Injury 46:975–984 25817163 10.1016/j.injury.2015.03.028

[CR53] Heerboth S, Lapinska K, Snyder N, Leary M, Rollinson S, Sarkar S (2014) Use of epigenetic drugs in disease: an overview. Genet Epigenet 6:9 25512710 10.4137/GEG.S12270 PMC4251063

[CR54] Hino Y, Nagaoka K, Oki S, Etoh K, Hino S, Nakao M (2022) Mitochondrial stress induces AREG expression and epigenomic remodeling through c-JUN and YAP-mediated enhancer activation. Nucleic Acids Res 50:9765–9779 36095121 10.1093/nar/gkac735 PMC9508833

[CR55] Hou Y, Lautrup S, Cordonnier S, Wang Y, Croteau DL, Zavala E, Zhang Y, Moritoh K, O’Connell JF, Baptiste BA et al (2018) NAD+ supplementation normalizes key Alzheimer’s features and DNA damage responses in a new AD mouse model with introduced DNA repair deficiency. Proc Natl Acad Sci USA 115:E1876–E1885 29432159 10.1073/pnas.1718819115 PMC5828618

[CR56] Imai SI, Guarente L (2014) NAD+ and sirtuins in aging and disease. Trends Cell Biol 24:464–471 24786309 10.1016/j.tcb.2014.04.002 PMC4112140

[CR57] Jackson SP, Bartek J (2009) The DNA-damage response in human biology and disease. Nature 461:1071–1078 19847258 10.1038/nature08467 PMC2906700

[CR58] Janssens S, Tschopp J (2006) Signals from within: the DNA-damage-induced NF-kappaB response. Cell Death Differ 13:773–784 16410802 10.1038/sj.cdd.4401843

[CR59] Jeong J, Juhn K, Lee H, Kim SH, Min BH, Lee KM, Cho MH, Park GH, Lee KH (2007) SIRT1 promotes DNA repair activity and deacetylation of Ku70. Exp Mol Med 39:8–13 17334224 10.1038/emm.2007.2

[CR60] Kaczmarska Z, Ortega E, Goudarzi A, Huang H, Kim S, Márquez JA, Zhao Y, Khochbin S, Panne D (2017) Structure of p300 in complex with acyl-CoA variants. Nat Chem Biol 13:21–29 27820805 10.1038/nchembio.2217 PMC5757799

[CR61] Kaelin WG, McKnight SL (2013) Influence of metabolism on epigenetics and disease. Cell 153:56–69 23540690 10.1016/j.cell.2013.03.004 PMC3775362

[CR62] Kim MB, Lee J, Lee JY (2024) Targeting mitochondrial dysfunction for the prevention and treatment of metabolic disease by bioactive food components. J Lipid Atheroscler 13:306 39355406 10.12997/jla.2024.13.3.306 PMC11439752

[CR63] Koeck T, Olsson AH, Nitert MD, Sharoyko VV, Ladenvall C, Kotova O, Reiling E, Rönn T, Parikh H, Taneera J et al (2011) A common variant in TFB1M is associated with reduced insulin secretion and increased future risk of type 2 diabetes. Cell Metab 13:80–91 21195351 10.1016/j.cmet.2010.12.007

[CR64] Kriaucionis S, Paterson A, Curtis J, Guy J, MacLeod N, Bird A (2006) Gene expression analysis exposes mitochondrial abnormalities in a mouse model of Rett syndrome. Mol Cell Biol 26:5033–5042 16782889 10.1128/MCB.01665-05 PMC1489175

[CR65] Kumar R, Zemaitis KJ, Fulcher JM, Paša-Tolić L (2024) Advances in mass spectrometry-enabled at single-cell resolution. Curr Opin Biotechnol 87:103096 10.1016/j.copbio.2024.103096 PMC12922598 38432187

[CR66] Kwon WY, Suh GJ, Jung YS, Park SM, Oh S, Kim SH, Rum Lee A, Kim JY, Kim H, Kim KA et al (2021) Circulating mitochondrial N-formyl peptides contribute to secondary nosocomial infection in patients with septic shock. Proc Natl Acad Sci USA 118:e2018538118 33888581 10.1073/pnas.2018538118 PMC8092466

[CR67] Lagger S, Connelly JC, Schweikert G, Webb S, Selfridge J, Ramsahoye BH, Yu M, He C, Sanguinetti G, Sowers LC et al (2017) MeCP2 recognizes cytosine methylated tri-nucleotide and di-nucleotide sequences to tune transcription in the mammalian brain. PLoS Genet 13:e1006793 10.1371/journal.pgen.1006793 PMC5446194 28498846

[CR68] Lane N, Martin W (2010) The energetics of genome complexity. Nature 467:929–934 20962839 10.1038/nature09486

[CR69] Laranjeira AC, Berger S, Kohlbrenner T, Greter NR, Hajnal A (2024) Nutritional vitamin B12 regulates RAS/MAPK-mediated cell fate decisions through one-carbon metabolism. Nat Commun 15:8178 10.1038/s41467-024-52556-3 PMC11408588 39289374

[CR70] Larsson NG, Wang J, Wilhelmsson H, Oldfors A, Rustin P, Lewandoski M, Barsh GS, Clayton DA (1998) Mitochondrial transcription factor A is necessary for mtDNA maintenance and embryogenesis in mice. Nat Genet 18:231–236 9500544 10.1038/ng0398-231

[CR71] Laukka T, Mariani CJ, Ihantola T, Cao JZ, Hokkanen J, Kaelin WG, Godley LA, Koivunen P (2016) Fumarate and succinate regulate expression of hypoxia-inducible genes via TET enzymes. J Biol Chem 291:4256–4265 26703470 10.1074/jbc.M115.688762 PMC4759199

[CR72] Leanza L, Ferraro P, Reichard P, Bianchi V (2008) Metabolic interrelations within guanine deoxynucleotide pools for mitochondrial and nuclear DNA maintenance. J Biol Chem 283:16437–16445 18417473 10.1074/jbc.M801572200

[CR73] Leemhuis H, Packman LC, Nightingale KP, Hollfelder F (2008) The human histone acetyltransferase P/CAF is a promiscuous histone propionyltransferase. Chembiochem 9:499–503 18247445 10.1002/cbic.200700556

[CR74] Lehmann S, Loh SHY, Martins LM (2016) Enhancing NAD+ salvage metabolism is neuroprotective in a PINK1 model of Parkinson’s disease. Biol Open 6:141 10.1242/bio.022186 PMC5312101 28011627

[CR75] Lewis JD, Meehan RR, Henzel WJ, Maurer-Fogy I, Jeppesen P, Klein F, Bird A (1992) Purification, sequence, and cellular localization of a novel chromosomal protein that binds to methylated DNA. Cell 69:905–914 1606614 10.1016/0092-8674(92)90610-o

[CR76] Li J, Bonkowski MS, Moniot S, Zhang D, Hubbard BP, Ling AJY, Rajman LA, Qin B, Lou Z, Gorbunova V et al (2017) A conserved NAD+ binding pocket that regulates protein-protein interactions during aging. Science 355:1312–1317 28336669 10.1126/science.aad8242 PMC5456119

[CR77] Li J, Lu L, Liu L, Ren X, Chen J, Yin X, Xiao Y, Li J, Wei G, Huang H et al (2023) HDAC1/2/3 are major histone desuccinylases critical for promoter desuccinylation. Cell Discov 9:85 37580347 10.1038/s41421-023-00573-9 PMC10425439

[CR78] Li W, Long Q, Wu H, Zhou Y, Duan L, Yuan H, Ding Y, Huang Y, Wu Y, Huang J et al (2022) Nuclear localization of mitochondrial TCA cycle enzymes modulates pluripotency via histone acetylation. Nat Commun 13:7414 36460681 10.1038/s41467-022-35199-0 PMC9718843

[CR79] Li Y, Zhao D, Chen Z, Li H (2016) YEATS domain: linking histone crotonylation to gene regulation. Transcription 8:9 27661789 10.1080/21541264.2016.1239602 PMC5402991

[CR80] Litonin D, Sologub M, Shi Y, Savkina M, Anikin M, Falkenberg M, Gustafsson CM, Temiakov D (2010) Human mitochondrial transcription revisited. J Biol Chem 285:18129–18133 20410300 10.1074/jbc.C110.128918 PMC2881736

[CR81] Liu H, Zhen C, Xie J, Luo Z, Zeng L, Zhao G, Lu S, Zhuang H, Fan H, Li X et al (2024) TFAM is an autophagy receptor that limits inflammation by binding to cytoplasmic mitochondrial DNA. Nat Cell Biol 26:878–891 38783142 10.1038/s41556-024-01419-6

[CR82] Liu J, Shangguan Y, Tang D, Dai Y (2021) Histone succinylation and its function on the nucleosome. J Cell Mol Med 25:7101 34160884 10.1111/jcmm.16676 PMC8335665

[CR83] Liu T, Chen X, Wei Z, Han X, Liu Y, Ma Z, Xia T, Gu X (2023) PPARα agonist fenofibrate prevents postoperative cognitive dysfunction by enhancing fatty acid oxidation in mice. Transl Neurosci 14:20220317 38023298 10.1515/tnsci-2022-0317 PMC10656729

[CR84] Liu X, Shen S, Wu P, Li F, Liu X, Wang C, Gong Q, Wu J, Yao X, Zhang H et al (2019) Structural insights into dimethylation of 12S rRNA by TFB1M: indispensable role in translation of mitochondrial genes and mitochondrial function. Nucleic Acids Res 47:7648–7665 31251801 10.1093/nar/gkz505 PMC6698656

[CR85] Löffler M, Jöckel J, Schuster G, Becker C (1997) Dihydroorotat-ubiquinone oxidoreductase links mitochondria in the biosynthesis of pyrimidine nucleotides. Mol Cell Biochem 174:125–129 9309676

[CR86] Lombardi AA, Gibb AA, Arif E, Kolmetzky DW, Tomar D, Luongo TS, Jadiya P, Murray EK, Lorkiewicz PK, Hajnóczky G et al (2019) Mitochondrial calcium exchange links metabolism with the epigenome to control cellular differentiation. Nat Commun 10:4509 10.1038/s41467-019-12103-x PMC6778142 31586055

[CR87] Lu Y, Chen Y, Hu W, Wang M, Wen X, Yang J (2023) Inhibition of ACSS2 attenuates alcoholic liver steatosis via epigenetically regulating de novo lipogenesis. Liver Int 43:1729–1740 37183518 10.1111/liv.15600

[CR88] Lyst MJ, Ekiert R, Ebert DH, Merusi C, Nowak J, Selfridge J, Guy J, Kastan NR, Robinson ND, De Lima Alves F et al (2013) Rett syndrome mutations abolish the interaction of MeCP2 with the NCoR/SMRT co-repressor. Nat Neurosci 16:898–902 23770565 10.1038/nn.3434 PMC3786392

[CR89] Ma S, Ming Y, Wu J, Cui G (2024) Cellular metabolism regulates the differentiation and function of T-cell subsets. Cell Mol Immunol 21:419–435 38565887 10.1038/s41423-024-01148-8 PMC11061161

[CR90] Majeed Y, Halabi N, Madani AY, Engelke R, Bhagwat AM, Abdesselem H, Agha MV, Vakayil M, Courjaret R, Goswami N et al (2021) SIRT1 promotes lipid metabolism and mitochondrial biogenesis in adipocytes and coordinates adipogenesis by targeting key enzymatic pathways. Sci Rep 11:8177 10.1038/s41598-021-87759-x PMC8046990 33854178

[CR91] Maksimovic I, David Y (2021) Non-enzymatic covalent modifications as a new chapter in the histone code. Trends Biochem Sci 46:718–730 33965314 10.1016/j.tibs.2021.04.004 PMC8364488

[CR92] Mao Y, Xia W, Jiang P (2026) Metabolites as signalling molecules in the tumour immune microenvironment. Nat Rev Immunol 26:422–438 10.1038/s41577-025-01258-y 41526478

[CR93] Martínez-Reyes I, Chandel NS (2020) Mitochondrial TCA cycle metabolites control physiology and disease. Nat Commun 11:102 10.1038/s41467-019-13668-3 PMC6941980 31900386

[CR94] McCool KW, Miyamoto S (2012) DNA damage-dependent NF-κB activation: NEMO turns nuclear signaling inside out. Immunol Rev 246:311–326 22435563 10.1111/j.1600-065X.2012.01101.x PMC3311051

[CR95] Medina CB, Mehrotra P, Arandjelovic S, Perry JSA, Guo Y, Morioka S, Barron B, Walk SF, Ghesquière B, Krupnick AS et al (2020) Metabolites released from apoptotic cells act as tissue messengers. Nature 580:130–135 32238926 10.1038/s41586-020-2121-3 PMC7217709

[CR96] Mei J, Ding P, Gao C, Zhou J, Li Z, Zhang C, Gao J (2025) Mitochondrial diseases: molecular pathogenesis and therapeutic advances. MedComm 6:e70385 40949487 10.1002/mco2.70385 PMC12426487

[CR97] Mentch SJ, Locasale JW (2016) One-carbon metabolism and epigenetics: understanding the specificity. Ann N Y Acad Sci 1363:91–98 26647078 10.1111/nyas.12956 PMC4801744

[CR98] Mentch SJ, Mehrmohamadi M, Huang L, Liu X, Gupta D, Mattocks D, Gómez Padilla P, Ables G, Bamman MM, Thalacker-Mercer AE et al (2015) Histone methylation dynamics and gene regulation occur through the sensing of one-carbon metabolism. Cell Metab 22:861–873 26411344 10.1016/j.cmet.2015.08.024 PMC4635069

[CR99] Mick E, Titov DV, Skinner OS, Sharma R, Jourdain AA, Mootha VK (2020) Distinct mitochondrial defects trigger the integrated stress response depending on the metabolic state of the cell. eLife 9:e49178 10.7554/eLife.49178 PMC7255802 32463360

[CR100] Molina-Granada D, González-Vioque E, Dibley MG, Cabrera-Pérez R, Vallbona-Garcia A, Torres-Torronteras J, Sazanov LA, Ryan MT, Cámara Y, Martí R (2022) Most mitochondrial dGTP is tightly bound to respiratory complex I through the NDUFA10 subunit. Commun Biol 5:620 10.1038/s42003-022-03568-6 PMC9226000 35739187

[CR101] Mondal A, Chakraborty A, Nandi S, Singh V, Kamat SS, Das C (2026) Transcription factor 19 modulates fatty acid elongation and unfolded protein response to attenuate palmitic acid-induced hepatic dysfunction. Nat Commun 17:5596 10.1038/s41467-026-72138-9 PMC13315908 42020413

[CR102] Morrish F, Noonan J, Perez-Olsen C, Gafken PR, Fitzgibbon M, Kelleher J, VanGilst M, Hockenbery D (2010) Myc-dependent mitochondrial generation of acetyl-CoA contributes to fatty acid biosynthesis and histone acetylation during cell cycle entry. J Biol Chem 285:36267 20813845 10.1074/jbc.M110.141606 PMC2978554

[CR103] Mottis A, Herzig S, Auwerx J (2019) Mitocellular communication: shaping health and disease. Science 366:827–832 31727828 10.1126/science.aax3768

[CR104] Nadalutti CA, Ayala-Peña S, Santos JH (2022) Mitochondrial DNA damage as driver of cellular outcomes. Am J Physiol Cell Physiol 322:C136–C150 34936503 10.1152/ajpcell.00389.2021 PMC8799395

[CR105] Nagaraj R, Sharpley MS, Chi F, Braas D, Zhou Y, Kim R, Clark AT, Banerjee U (2017) Nuclear localization of mitochondrial TCA cycle enzymes as a critical step in mammalian zygotic genome activation. Cell 168:210–223.e11 28086092 10.1016/j.cell.2016.12.026 PMC5321559

[CR106] Nikkanen J, Forsström S, Euro L, Paetau I, Kohnz RA, Wang L, Chilov D, Viinamäki J, Roivainen A, Marjamäki P et al (2016) Mitochondrial DNA replication defects disturb cellular dNTP pools and remodel one-carbon metabolism. Cell Metab 23:635–648 26924217 10.1016/j.cmet.2016.01.019

[CR107] Nissanka N, Moraes CT (2018) Mitochondrial DNA damage and reactive oxygen species in neurodegenerative disease. FEBS Lett 592:728–742 29281123 10.1002/1873-3468.12956 PMC6942696

[CR108] Nshanian M, Gruber JJ, Geller BS, Chleilat F, Lancaster SM, White SM, Alexandrova L, Camarillo JM, Kelleher NL, Zhao Y et al (2025) Short-chain fatty acid metabolites propionate and butyrate are unique epigenetic regulatory elements linking diet, metabolism and gene expression. Nat Metab 7:196–211 39789354 10.1038/s42255-024-01191-9 PMC11774759

[CR109] Nunnari J, Suomalainen A (2012) Mitochondria: in sickness and in health. Cell 148:1145–1159 22424226 10.1016/j.cell.2012.02.035 PMC5381524

[CR110] Oanh NTK, Lee HS, Kim YH, Min S, Park YJ, Heo J, Park YY, Lim WC, Cho H (2022) Regulation of nuclear DNA damage response by mitochondrial morphofunctional pathway. Nucleic Acids Res 50:9247–9259 35979947 10.1093/nar/gkac690 PMC9458461

[CR111] Pankowicz FP, Barzi M, Legras X, Hubert L, Mi T, Tomolonis JA, Ravishankar M, Sun Q, Yang D, Borowiak M et al (2016) Reprogramming metabolic pathways in vivo with CRISPR/Cas9 genome editing to treat hereditary tyrosinaemia. Nat Commun 7:12642 10.1038/ncomms12642 PMC5013601 27572891

[CR112] Papsdorf K, Brunet A (2019) Linking lipid metabolism to chromatin regulation in aging. Trends Cell Biol 29:97–116 30316636 10.1016/j.tcb.2018.09.004 PMC6340780

[CR113] Patananan AN, Wu TH, Chiou PY, Teitell MA (2016) Modifying the mitochondrial genome. Cell Metab 23:785 27166943 10.1016/j.cmet.2016.04.004 PMC4864607

[CR114] Petrova B, Maynard AG, Wang P, Kanarek N (2023) Regulatory mechanisms of one-carbon metabolism enzymes. J Biol Chem 299:105457 10.1016/j.jbc.2023.105457 PMC10758965 37949226

[CR115] Pfanner N, Warscheid B, Wiedemann N (2019) Mitochondrial proteins: from biogenesis to functional networks. Nat Rev Mol Cell Biol 20:267–284 30626975 10.1038/s41580-018-0092-0 PMC6684368

[CR116] Pietrocola F, Galluzzi L, Bravo-San Pedro JM, Madeo F, Kroemer G (2015) Acetyl coenzyme A: a central metabolite and second messenger. Cell Metab 21:805–821 26039447 10.1016/j.cmet.2015.05.014

[CR117] Pontarin G, Gallinaro L, Ferraro P, Reichard P, Bianchi V (2003) Origins of mitochondrial thymidine triphosphate: dynamic relations to cytosolic pools. Proc Natl Acad Sci USA 100:12159–12164 14519855 10.1073/pnas.1635259100 PMC218729

[CR118] Qian, Kumar, Roginskaya, Fouquerel, Opresko PL, Shiva S, Watkins SC, Kolodieznyi D, Bruchez MP, Van Houten B (2019) Chemoptogenetic damage to mitochondria causes rapid telomere dysfunction. Proc Natl Acad Sci USA 116:18435–18444 31451640 10.1073/pnas.1910574116 PMC6744920

[CR119] Quirós PM, Mottis A, Auwerx J (2016) Mitonuclear communication in homeostasis and stress. Nat Rev Mol Cell Biol 17:213–226 26956194 10.1038/nrm.2016.23

[CR120] Ramachandran A, Basu U, Sultana S, Nandakumar D, Patel SS (2016) Human mitochondrial transcription factors TFAM and TFB2M work synergistically in promoter melting during transcription initiation. Nucleic Acids Res 45:861 27903899 10.1093/nar/gkw1157 PMC5314767

[CR121] Rawls J, Knecht W, Diekert K, Lill R, Löffler M (2000) Requirements for the mitochondrial import and localization of dihydroorotate dehydrogenase. Eur J Biochem 267:2079–2087 10727948 10.1046/j.1432-1327.2000.01213.x

[CR122] Rizvi SZ, Chan WS, Maino E, Steiman S, Forguson G, Klepfish M, Cohn RD, Ivakine EA (2024) Multi-gene duplication removal in an engineered human cellular MECP2 duplication syndrome model with an IRAK1-MECP2 duplication. Mol Ther Nucleic Acids 35:102356 10.1016/j.omtn.2024.102356 PMC11539574 39507402

[CR123] Roberti M, Polosa PL, Bruni F, Manzari C, Deceglie S, Gadaleta MN, Cantatore P (2009) The MTERF family proteins: mitochondrial transcription regulators and beyond. Biochim Biophys Acta Bioenerg 1787:303–311 10.1016/j.bbabio.2009.01.013 19366610

[CR124] Rose NR, Mc Donough MA, King ONF, Kawamura A, Schofield CJ (2011) Inhibition of 2-oxoglutarate dependent oxygenases. Chem Soc Rev 40:4364–4397 21390379 10.1039/c0cs00203h

[CR125] Roy Chowdhury A, Srinivasan S, Csordás G, Hajnóczky G, Avadhani NG (2020) Dysregulation of RyR calcium channel causes the onset of mitochondrial retrograde signaling. iScience 23:101370 32738613 10.1016/j.isci.2020.101370 PMC7394923

[CR126] Rutten MGS, Rots MG, Oosterveer MH (2019) Exploiting epigenetics for the treatment of inborn errors of metabolism. J Inherit Metab Dis 43:63 30916397 10.1002/jimd.12093 PMC7041640

[CR127] Sabari BR, Tang Z, Huang H, Yong-Gonzalez V, Molina H, Kong HE, Dai L, Shimada M, Cross JR, Zhao Y et al (2015) Intracellular crotonyl-CoA stimulates transcription through p300-catalyzed histone crotonylation. Mol Cell 58:203–215 25818647 10.1016/j.molcel.2015.02.029 PMC4501262

[CR128] Sahu V, Lu C (2025) Metabolism-driven chromatin dynamics: molecular principles and technological advances. Mol Cell 85:262–275 39824167 10.1016/j.molcel.2024.12.012 PMC11750176

[CR129] Santos JH (2021) Mitochondria signaling to the epigenome: a novel role for an old organelle. Free Radic Biol Med 170:59–69 33271282 10.1016/j.freeradbiomed.2020.11.016 PMC8166959

[CR130] Scarpulla RC (2006) Nuclear control of respiratory gene expression in mammalian cells. J Cell Biochem 97:673–683 16329141 10.1002/jcb.20743

[CR131] Scarpulla RC (2008) Transcriptional paradigms in mammalian mitochondrial biogenesis and function. Physiol Rev 88:611–638 18391175 10.1152/physrev.00025.2007

[CR132] Scheibye-Knudsen M, Fang EF, Croteau DL, Wilson DM, Bohr VA (2015) Protecting the mitochondrial powerhouse. Trends Cell Biol 25:158–170 25499735 10.1016/j.tcb.2014.11.002 PMC5576887

[CR133] Scheibye-Knudsen M, Ramamoorthy M, Sykora P, Maynard S, Lin PC, Minor RK, Wilson DM, Cooper M, Spencer R, de Cabo R et al (2012) Cockayne syndrome group B protein prevents the accumulation of damaged mitochondria by promoting mitochondrial autophagy. J Exp Med 209:855–869 22473955 10.1084/jem.20111721 PMC3328359

[CR134] Sciacovelli M, Gonçalves E, Johnson TI, Zecchini VR, Da Costa ASH, Gaude E, Drubbel AV, Theobald SJ, Abbo SR, Tran MGB et al (2016) Fumarate is an epigenetic modifier that elicits epithelial-to-mesenchymal transition. Nature 537:544–547 27580029 10.1038/nature19353 PMC5136292

[CR135] Sena LA, Chandel NS (2012) Physiological roles of mitochondrial reactive oxygen species. Mol Cell 48:158–167 23102266 10.1016/j.molcel.2012.09.025 PMC3484374

[CR136] Shi L, Tu BP (2015) Acetyl-CoA and the regulation of metabolism: mechanisms and consequences. Curr Opin Cell Biol 33:125–131 25703630 10.1016/j.ceb.2015.02.003 PMC4380630

[CR137] Shi Y, Lan F, Matson C, Mulligan P, Whetstine JR, Cole PA, Casero RA, Shi Y (2004) Histone demethylation mediated by the nuclear amine oxidase homolog LSD1. Cell 119:941–953 15620353 10.1016/j.cell.2004.12.012

[CR138] Shiloh Y, Ziv Y (2013) The ATM protein kinase: regulating the cellular response to genotoxic stress, and more. Nat Rev Mol Cell Biol 14:197–210 23486281 10.1038/nrm3546

[CR139] Shimazu T, Hirschey MD, Newman J, He W, Shirakawa K, Le Moan N, Grueter CA, Lim H, Saunders LR, Stevens RD et al (2013) Suppression of oxidative stress by β-hydroxybutyrate, an endogenous histone deacetylase inhibitor. Science 339:211–214 23223453 10.1126/science.1227166 PMC3735349

[CR140] Shyh-Chang N, Locasale JW, Lyssiotis CA, Zheng Y, Teo RY, Ratanasirintrawoot S, Zhang J, Onder T, Unternaehrer JJ, Zhu H et al (2013) Influence of threonine metabolism on S-adenosylmethionine and histone methylation. Science 339:222–226 23118012 10.1126/science.1226603 PMC3652341

[CR141] Signorini C, De Felice C, Leoncini S, Møller RS, Zollo G, Buoni S, Cortelazzo A, Guerranti R, Durand T, Ciccoli L et al (2016) MECP2 duplication syndrome: evidence of enhanced oxidative stress. a comparison with Rett syndrome. PLoS ONE 11:e0150101 26930212 10.1371/journal.pone.0150101 PMC4773238

[CR142] Singh KK (2004) Mitochondria damage checkpoint in apoptosis and genome stability. FEMS Yeast Res 5:127–132 15489195 10.1016/j.femsyr.2004.04.008

[CR143] Sivanand S, Viney I, Wellen KE (2018) Spatiotemporal control of acetyl-CoA metabolism in chromatin regulation. Trends Biochem Sci 43:61–74 29174173 10.1016/j.tibs.2017.11.004 PMC5741483

[CR144] Song S, Wheeler LJ, Mathews CK (2003) Deoxyribonucleotide pool imbalance stimulates deletions in hela cell mitochondrial DNA. J Biol Chem 278:43893–43896 13679382 10.1074/jbc.C300401200

[CR145] Su X, Wellen KE, Rabinowitz JD (2016) Metabolic control of methylation and acetylation. Curr Opin Chem Biol 30:52–60 26629854 10.1016/j.cbpa.2015.10.030 PMC4731252

[CR146] Sullivan LB, Gui DY, Hosios AM, Bush LN, Freinkman E, Vander Heiden MG (2015) Supporting aspartate biosynthesis is an essential function of respiration in proliferating. Cells Cell 162:552–563 26232225 10.1016/j.cell.2015.07.017 PMC4522278

[CR147] Sun C, Wang A, Zhou Y, Chen P, Wang X, Huang J, Gao J, Wang X, Shu L, Lu J et al (2023) Spatially resolved multi-omics highlights cell-specific metabolic remodeling and interactions in gastric cancer. Nat Commun 14:2692 10.1038/s41467-023-38360-5 PMC10172194 37164975

[CR148] Sutendra G, Kinnaird A, Dromparis P, Paulin R, Stenson TH, Haromy A, Hashimoto K, Zhang N, Flaim E, Michelakis ED (2014) A nuclear pyruvate dehydrogenase complex is important for the generation of acetyl-CoA and histone acetylation. Cell 158:84–97 24995980 10.1016/j.cell.2014.04.046

[CR149] Sykora P, Kanno S, Akbari M, Kulikowicz T, Baptiste BA, Leandro GS, Lu H, Tian J, May A, Becker KA et al (2017) DNA polymerase beta participates in mitochondrial DNA repair. Mol Cell Biol 37:e00237–17 28559431 10.1128/MCB.00237-17 PMC5533889

[CR150] Tatar M, Sedivy JM (2016) Mitochondria: masters of epigenetics. Cell 165:1052–1054 27203109 10.1016/j.cell.2016.05.021 PMC5383427

[CR151] Taylor MJ, Lukowski JK, Anderton CR (2021) Spatially resolved mass spectrometry at the single cell: recent innovations in proteomics and metabolomics. J Am Soc Mass Spectrom 32:872–894 33656885 10.1021/jasms.0c00439 PMC8033567

[CR152] Tian Y, Garcia G, Bian Q, Steffen KK, Joe L, Wolff S, Meyer BJ, Dillin A (2016) Mitochondrial stress induces chromatin reorganization to promote longevity and UPRmt. Cell 165:1197–1208 27133166 10.1016/j.cell.2016.04.011 PMC4889216

[CR153] Tsukada YI, Fang J, Erdjument-Bromage H, Warren ME, Borchers CH, Tempst P, Zhang Y (2006) Histone demethylation by a family of JmjC domain-containing proteins. Nature 439:811–816 16362057 10.1038/nature04433

[CR154] Ulanovskaya OA, Zuhl AM, Cravatt BF (2013) NNMT promotes epigenetic remodeling in cancer by creating a metabolic methylation sink. Nat Chem Biol 9:300–306 23455543 10.1038/nchembio.1204 PMC3631284

[CR155] Valentin-Vega YA, MacLean KH, Tait-Mulder J, Milasta S, Steeves M, Dorsey FC, Cleveland JL, Green DR, Kastan MB (2012) Mitochondrial dysfunction in ataxia-telangiectasia. Blood 119:1490–1500 22144182 10.1182/blood-2011-08-373639 PMC3286212

[CR156] Veatch JR, McMurray MA, Nelson ZW, Gottschling DE (2009) Mitochondrial dysfunction leads to nuclear genome instability via an iron-sulfur cluster defect. Cell 137:1247–1258 19563757 10.1016/j.cell.2009.04.014 PMC2759275

[CR157] Verdin E (2015) NAD^+^ in aging, metabolism, and neurodegeneration. Science 350:1208–1213 26785480 10.1126/science.aac4854

[CR158] Walker EM, Pearson GL, Lawlor N, Stendahl AM, Lietzke A, Sidarala V, Zhu J, Stromer T, Reck EC, Li J et al (2025) Retrograde mitochondrial signaling governs the identity and maturity of metabolic tissues. Science 388:eadf2034 10.1126/science.adf2034 PMC11985298 39913641

[CR159] Wallace DC (1999) Mitochondrial diseases in man and mouse. Science 283:1482–1488 10066162 10.1126/science.283.5407.1482

[CR160] Wallace DC (2005) A mitochondrial paradigm of metabolic and degenerative diseases, aging, and cancer: a dawn for evolutionary medicine. Annu Rev Genet 39:359–407 16285865 10.1146/annurev.genet.39.110304.095751 PMC2821041

[CR161] Wang Y, Guo YR, Liu K, Yin Z, Liu R, Xia Y, Tan L, Yang P, Lee JH, Li XJ et al (2017) KAT2A coupled with the α-KGDH complex acts as a histone H3 succinyltransferase. Nature 552:273–277 29211711 10.1038/nature25003 PMC5841452

[CR162] Wang YM, Wu Y, Zheng YF, Wang HY (2021) MeCP2 duplication causes hyperandrogenism by upregulating LHCGR and downregulating RORα. Cell Death Dis 12:999 34697294 10.1038/s41419-021-04277-4 PMC8545957

[CR163] Wellen KE, Hatzivassiliou G, Sachdeva UM, Bui TV, Cross JR, Thompson CB (2009) ATP-citrate lyase links cellular metabolism to histone acetylation. Science 324:1076–1080 19461003 10.1126/science.1164097 PMC2746744

[CR164] Wiese M, Bannister AJ (2020) Two genomes, one cell: Mitochondrial-nuclear coordination via epigenetic pathways. Mol Metab 38:100942 10.1016/j.molmet.2020.01.006 PMC7300384 32217072

[CR165] Wu X, Zhang Y (2017) TET-mediated active DNA demethylation: mechanism, function and beyond. Nat Rev Genet 18:517–534 28555658 10.1038/nrg.2017.33

[CR166] Wu Z, Oeck S, West AP, Mangalhara KC, Sainz AG, Newman LE, Zhang XO, Wu L, Yan Q, Bosenberg M et al (2019) Mitochondrial DNA stress signalling protects the nuclear. Genome Nat Metab 1:1209–1218 32395698 10.1038/s42255-019-0150-8 PMC7213273

[CR167] Wu Z, Sainz AG, Shadel GS (2021) Mitochondrial DNA: cellular genotoxic stress sentinel. Trends Biochem Sci 46:812–821 34088564 10.1016/j.tibs.2021.05.004 PMC9809014

[CR168] Xiao M, Yang H, Xu W, Ma S, Lin H, Zhu H, Liu L, Liu Y, Yang C, Xu Y et al (2012) Inhibition of α-KG-dependent histone and DNA demethylases by fumarate and succinate that are accumulated in mutations of FH and SDH tumor suppressors. Genes Dev 26:1326–1338 22677546 10.1101/gad.191056.112 PMC3387660

[CR169] Xu W, Yang H, Liu Y, Yang Y, Wang P, Kim SH, Ito S, Yang C, Wang P, Xiao MT et al (2011) Oncometabolite 2-hydroxyglutarate is a competitive inhibitor of α-ketoglutarate-dependent dioxygenases. Cancer Cell 19:17 21251613 10.1016/j.ccr.2010.12.014 PMC3229304

[CR170] Yang D, Oyaizu Y, Oyaizu H, Olsen GJ, Woese CR (1985) Mitochondrial origins. Proc Natl Acad Sci USA 82:4443–4447 3892535 10.1073/pnas.82.13.4443 PMC391117

[CR171] Yang D, Wu X, Yao Y, Duan M, Wang X, Li G, Guo A, Wu M, Liu Y, Zheng J et al (2024) An RNA editing strategy rescues gene duplication in a mouse model of MECP2 duplication syndrome and nonhuman primates. Nat Neurosci 28:72–83 39668251 10.1038/s41593-024-01838-6

[CR172] Yuan Y, Zhu C, Wang Y, Sun J, Feng J, Ma Z, Li P, Peng W, Yin C, Xu G et al (2022) α-Ketoglutaric acid ameliorates hyperglycemia in diabetes by inhibiting hepatic gluconeogenesis via serpina1e signaling. Sci Adv 8:eabn2879 35507647 10.1126/sciadv.abn2879 PMC9067931

[CR173] Zhang Q, Raoof M, Chen Y, Sumi Y, Sursal T, Junger W, Brohi K, Itagaki K, Hauser CJ (2010) Circulating mitochondrial DAMPs cause inflammatory responses to injury. Nature 464:104–107 20203610 10.1038/nature08780 PMC2843437

[CR174] Zhou BBS, Elledge SJ (2000) The DNA damage response: putting checkpoints in perspective. Nature 408:433–439 11100718 10.1038/35044005

