# Supplementary material for: Metabolites with a message: impacts on epigenetics and implications for epimetabopathies
Source: EMBO Rep. 2026 Jul 29;27(17):4969–85. doi: 10.1038/s44319-026-00891-5 (PMC13554220; doi:10.1038/s44319-026-00891-5)
Supplement: Supplementary file 1 [file 44319_2026_891_MOESM1_ESM.pdf]

EMBO reports

METABOLITES WITH A MESSAGE: IMPACTS ON EPIGENETICS AND IMPLICATIONS FOR EPIMETABOPATHIES

Indrakshi Banerjee, Atanu Mondal, Siddhesh Kamat, and Chandrima Das

Corresponding author(s): Siddhesh Kamat (siddhesh@iiserpune.ac.in) , Chandrima Das

(chandrima.das@saha.ac.in)

Review Timeline:

Submission Date:	1st May 26
Editorial Decision:	10th Jun 26
Revision Received:	2nd Jul 26
Accepted:	21st Jul 26

Editor: Esther Schnapp

Transaction Report:

(Note: With the exception of the correction of typographical or spelling errors that could be a source of ambiguity, letters and reports are not edited. Depending on transfer agreements, referee reports obtained elsewhere may or may not be included in this compilation. Referee reports are anonymous unless the Referee chooses to sign their reports.)

Dear Prof. Kamat,

Thank you for the submission of your Review to EMBO reports. Below you will find the set of comments we have now received on your manuscript.

As you will see, all referees acknowledge that the review topic is of general interest and timely, which is great. However, from the comments, especially from referee 1, it also appears that the review is a very borderline case, because it is not coherent but seems to touch different topics that are not always well connected or worked out. This is a difficult and important issue to address, and I think it is worth trying, given the overall interest. I would like to suggest that you address referee 1's concerns (as well as all other referee concerns) to the best of your abilities and we will then see how we can work with the revised review. Does this sound ok?

As referee 1 suggests, it will probably be good to focus the review on one (or max a few) main topics, and this could be how mitochondria and nuclear functions are coupled and integrated, as well as the relationship between the mitochondria, dNTP pools and genome stability.

If it is helpful, we can also discuss the review revisions in a video chat.

The text in Figure 3 needs to be more cautious, to stress that all these are potential effects.

Please also remove all overly general, non-concrete statements and flowery/poetic language.

As for timing, would it be possible for you to submit the revised review by July 10th? If you anticipate a problem meeting this deadline, then please just let me know. Please also co-submit a point-by-point response to all referee comments.

With best wishes,
Esther

Esther Schnapp, PhD
Senior Editor
EMBO reports

Referee #1:

This review tries to do too much - by trying to bridge multiple processes that are intracellular (mitochondria - nucleus), or systemic (across tissues and organs), and then relate this to metabopathies that affect epigenetic states in cells and across tissues. By doing so - a clear and coherent message does not emerge. There are many interesting ideas within this review, and each could do with a more detailed extraposition and attempt to synthesise. However, in its present form - this review does not synthesise ideas, and instead 'throws up' multiple ideas, none of which is fleshed out well, nor are their connections made clear. There are parts of this review that is excellent, and interesting, but this does not come together. In addition, there are some assumptions made that are somewhat incorrect. So, while this raises interesting ideas, it is hard to figure out what the end message is. The part that is more coherent is the highlighting that the mitochondria and nuclear function are deeply coupled and integrated, as well as the relationship between the mitochondria, dNTP pools and genome stability. This part (and mito - nuclear cross talk) could become the cornerstone of this review.

Some other points:
1. on mitochondrial TCA intermediate as substrates for acetyltransferases/demethylases - this is an over simplistic statement. The pools of mitochondrial acetylCoA and nucleo-cytosolic AcCoA are distinct, partitioned, and obtained differently (for example, much of the nuclear acetylation comes from AcetylCoA sourced from cytosolic acetate) etc.
2. The connection between DNA repair and mitochondria is presented vaguely - and cause/consequence connections aren't well fleshed out or thought through. This, if tightened up, can become a very interesting segment.
3. This cause/consequence confusion becomes more apparent in the current presentation of the nuclear-chromatin/mitochondrial cross-control (anterograde/retrograde). It is obvious that global gene expression state will respond to mitochondrial states, and vice versa - this is essential for cellular homeostasis, or a change in state. This will include components of redox, or co-factor depletion or ATP, or calcium etc. The fact that mitochondria are dynamic signalling hubs is now very clear. So it isn't very clear what the authors want to emphasise on.
When suddenly statements like "The mitochondria thereby work as epigenomic sculptors, imprinting stress into the chromatin as sturdy, durable epigenetic marks through a cascade of transient metabolic signalling." it is totally unclear how, or what the specific mechanisms are, and how this is related to future stress responses.
4. The transition to SAM and SAM metabolism, which is of course closely coupled to 1C-folate metabolism, is unclear. What

specific point do the authors want to make, related to SAM metabolism, mitochondria and nuclear/chromatin cross talk.
5. The relationship between succinate, fumarate, 2-OG and DNA/histone demethylases is very well known. But it is still unclear how changes in these pools of metabolites can lead to specific changes in patterns of gene expression. This is a widely contested area of research, where there are large gaps in quantitative information that is necessary. While I appreciate the idea, it is not clear what point can be made.
6. The links between fatty acid metabolism and histone modifications can be better fleshed out - to make a synthesising point. There is substantial, and growing literature in this space - particularly how this affects acetylation/succnylation/butyryltion programs etc. This section, if fleshed out well - in a more coherent reorganisation, can help bring this review together.

7. the section on circulating metabolites is a conceptual jump - it is very difficult to specifically link mitochondrial balance to systemic changes (after circulation) and from here move to what the authors describe as 'epimetabopathies'. From here, making a jump to phase-transitioning and phase separation, and chromatin/gene expression changes appears completely disjointed - and an enthusiastic expression of multiple possible ideas, but not a rigorous exposition.

On the figures - Figure 1 is terrific and comprehensive. Figure 3 is euphemistic - and impossible to present as established fact.

Referee #2:

This manuscript reviews a complex, yet very relevant topic: the cross-talk between mitochondrial metabolism and nuclear epigenetic processes. The manuscript first deals with the regulation of the nucleotide pools that serve both nuclear and mitochondrial DNA synthesis. Imbalance in one the two processes inevitably affects the other. This has profound effects on the ability of cells to repair DNA that is in turn related to cell cycle control and cell fate. Here, the manuscript discusses the role of SIRT1. The narrative mentions "SIRT1's synchronous activity". At this point, I was a bit lost as the meaning of "synchronous" in this context is not fully clear. A better explanation will help the reader. The manuscript continues by discussing anterograde and retrograde signallings. Anterograde signaling is mostly the regulated expression and function of transcription factors that control the expression of mitochondrial proteins. Retrograde signalling alerts the nucleus of a stressful condition of mitochondria. Here, the text mentions a change in H3K9 methylation. It would be interesting to describe, if known, how such a change is brought about and why it is specific for this methylation mark. This is the core of the review because one appreciates how mitochondrial metabolites can directly affect the activity of chromatin-modifying enzymes. Indeed, alterations in these processes are tumorigenic. At this point, the review mentions N-formylpeptides entering circulation and functioning as immunomodulators. This paragraph might be more logically moved to the section on "Circulating metabolites". The manuscript makes the point that alterations in these regulatory circuits can be pathogenic. The discussion of Rett syndrome is particularly illuminating in this regard. Time will tell if this knowledge can be translated into efficient pharmacological therapies.
Overall, I found this review useful and timely. Aside from the minor suggestions offered above, it can be published in this Journal.

Abstract
The sentence "Mitochondrial metabolites are the main directors of life's molecular ballet" is somewhat cryptic obscure and probably overstated.

Referee #3:

In this review article, the authors describe how mitochondrial metabolites can regulate epigenetics in physiological and disease conditions. In this lyrically written manuscript, the authors introduce a new term - "epimetabopathies" to describe this phenomenon. This topic is of great biomedical significance, because mitochondrial dysfunction has been associated with numerous common as well as rare genetic diseases. Moreover, environmental and dietary factors, such as high fat diet, is known to perturb mitochondrial function and cellular epigenetic landscape. The authors have nicely organized their article in different subtopics - anterograde vs retrograde signaling, circulating metabolites regulating systemic interactions, etc. However, their introduction of new terms without defining them, use of some cryptic sentences, a lack of mechanistic description, tangential forays into irrelevant areas, and occasional errors makes it a difficult read. Below I highlight, few such examples.
1. On page 5, the authors state that "mitochondria confiscate certain species of dNTPs". The authors can be more precise by identifying/listing the specific species of dNTPs.
2. The section "Tuning of DNA repair enzymes by mitochondria" appears a bit superficial without detailed mechanistic insights and explanations of studies discussed. For example, the sentence on page 7 "It has been observed that impaired mitochondrial function compromises both nuclear genome stability and DDR through depletion of metabolic cofactors essential for repair enzyme function (Nadalutti et al, 2022; Sykora et al, 2017; Hou et al, 2018)" the authors should elaborate on which cofactors are they talking about.
3. Again, on page 7, the authors should explain on how NAD+ connects to PARP?
4. The authors should describe how mitochondrial dysfunction perturbs NAD+ homeostasis, which in turn impacts epigenetic landscape.
5. The term "morphofunctional" or "dual-axis control" is confusing. It is best to avoid introducing jargons to ensure that the main message is not lost to the readers.
6. Define acronyms on first use, for example OXPHOS, page 4. On page 9, PCG-1 should be PGC-1.

7. It is not clear what the author means by "chromatin substrates?" Again, it is better to spell out what they mean.
9. The sentence "For example, extracellular metabolites like α-ketoglutarate (α-KG) can bind to the purinergic receptor, P2RX4, facilitating nuclear import of intracellular α-KG via SLC25A11" is very confusing, because SLC25A11 is a mitochondrial α-KG exporter and not a nuclear α-KG importer.

`REBUTTAL

We thank the reviewers for their careful and constructive review of our manuscript. We carefully considered all of the comments, insights, and suggestions of the reviewers. We have addressed them in their entirety. We hope that the reviewers find our rebuttal to suitably address their concerns.

- Reviewers' comments: *blue italic text*
- Our responses: black regular text
- Sample of text from revised manuscript = *green italic text*
- Table/Figure citations = red text

EDITORIAL COMMENTS

Comment 1: *As you will see, all referees acknowledge that the review topic is of general interest and timely, which is great.*

Response: We thank the Editor for this encouraging comment. We are pleased that all reviewers found the topic to be timely and of broad interest.

Comment 2: *However, from the comments, especially from referee 1, it also appears that the review is a very borderline case, because it is not coherent but seems to touch different topics that are not always well connected or worked out. This is a difficult and important issue to address, and I think it is worth trying, given the overall interest. I would like to suggest that you address referee 1's concerns (as well as all other referee concerns) to the best of your abilities and we will then see how we can work with the revised review. Does this sound ok?*

Response: We thank the Editor for this guidance. We have carefully considered Referee 1's concerns and revised the manuscript substantially to improve its overall organization and coherence. To improve clarity and better define the relationships between topics, we have introduced a hierarchical section numbering (e.g., 1, 1.1, 1.2, etc.), which can be modified as needed to comply with *EMBO Reports* formatting requirements. We have also addressed all comments raised by the reviewers to the best of our ability and hope that these revisions have considerably improved the quality of the review. We are grateful for the opportunity to revise the manuscript and appreciate the Editor's willingness to consider the revised version.

Comment 3: *As referee 1 suggests, it will probably be good to focus the review on one (or max a few) main topics, and this could be how mitochondria and nuclear functions are coupled and integrated, as well as the relationship between the mitochondria, dNTP pools and genome stability.*

Response: We appreciate the editor's suggestion and the recognition of the sections discussing mitochondrial-nuclear crosstalk and the role of mitochondria in regulating dNTP pools and genome stability. We have revised the manuscript to strengthen this central narrative and improve the integration of the different topics discussed. The sections on mitochondrial control of dNTP supply, DNA repair and bidirectional mitochondria-chromatin communication now more clearly establish the core framework of the review (**Section 2:** Mitochondria in Genome Surveillance,

beyond Powerhouse Duties, **Section 3.** Reciprocal Rewiring of Chromatin and Mitochondria, and **Section 4.** Metabolites inscribe stress signals into the epigenome).

The subsequent sections on systemic metabolic signalling and epimetabopathies are intended as extensions of this fundamental mitochondria-nucleus crosstalk to higher levels of biological organization and disease relevance. Importantly, these sections benefited significantly from the comments of Reviewers 2 and 3, who provided constructive suggestions to further improve their clarity, organisation and presentation. Accordingly, rather than removing these sections, we have revised and strengthened them, incorporating the reviewers' recommendations and more clearly framing them as extensions of the core mitochondria-nucleus crosstalk framework to broader physiological and disease contexts.

Comment 4: *The text in Figure 3 needs to be more cautious, to stress that all these are potential effects.*

Response: We thank the editor for this comment. We agree that the mechanisms depicted in Figure 3 constitute the proposed model of interorgan metabo-epigenetic communication, based on the sparse existing literature. Accordingly, we have revised Figure 3 and its legend to use more cautious language throughout and to emphasize the hypothetical nature of the proposed pathways. The revised figure and legend are as follows:

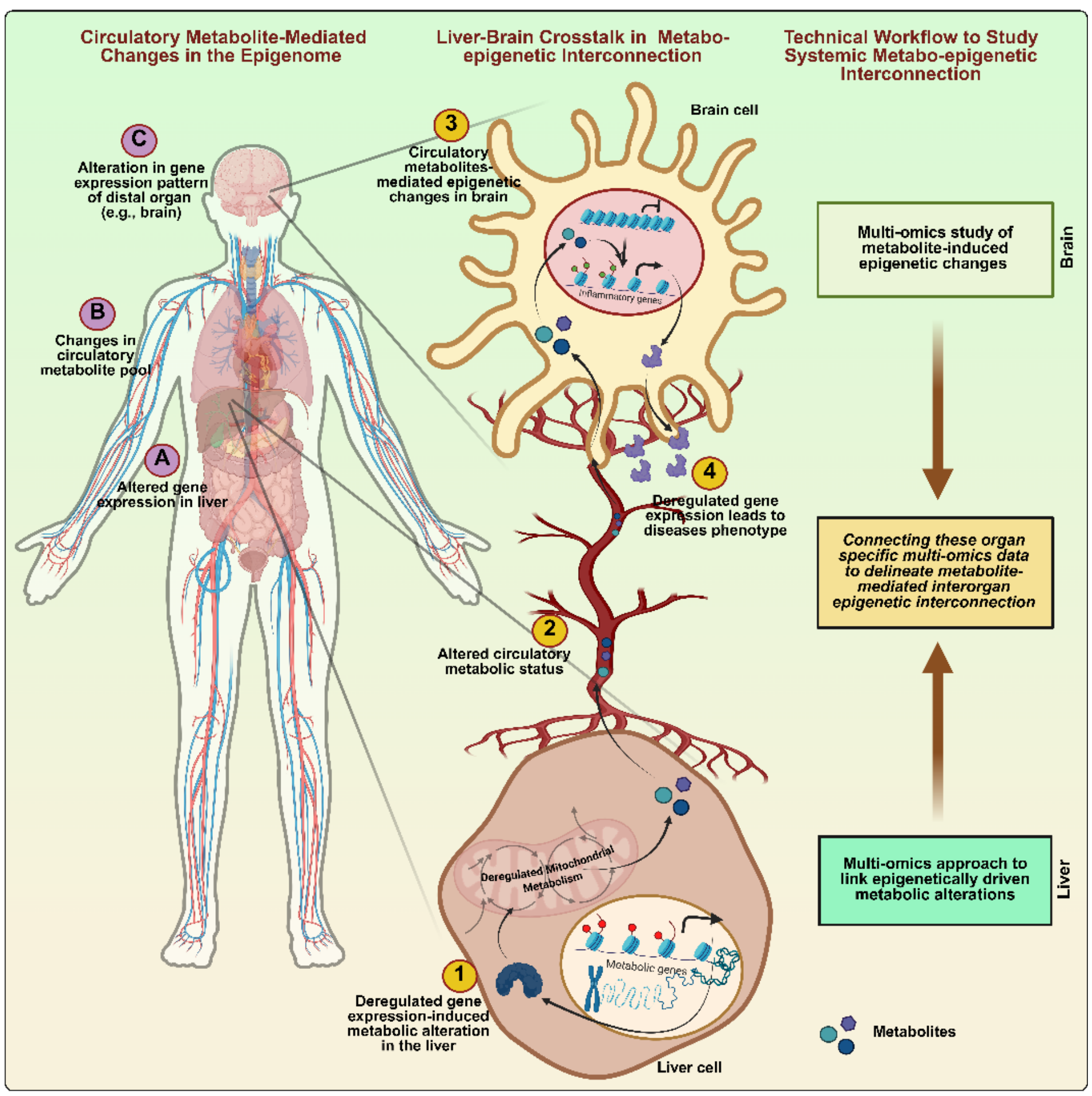

"***Figure 3. Proposed model of metabolite-mediated interorgan crosstalk and its potential contribution to epigenetic and metabolic reprogramming across the liver-brain axis.*** *Alterations in hepatic gene expression, which may occur under different dietary or metabolic conditions, can reshape the hepatic metabolic landscape and potentially alter the pool of circulating metabolites. These metabolic intermediates may reach distal organs like the brain, where they could cause epigenetic rewiring and changes in gene expression patterns, thereby contributing to disease-associated phenotypes. The left panel (A - C) illustrates putative systemic effects, while the middle panel (1 - 4) depicts proposed intercellular mechanisms. The epigenetic modifications depicted may regulate gene expression, but are yet to be explored in the context of metabolite-mediated interorgan crosstalk. The right panel highlights the proposed multi-omics approach that may facilitate the mapping of organ-specific epimetabolic states and help evaluate these inter-organ communication pathways. Created in BioRender. Das, C. (2026) https://BioRender.com/l3x7x4r.*"

Comment 5: *Please also remove all overly general, non-concrete statements and flowery/poetic language.*

Response: We thank the Editor for this suggestion. In the revised version, we have carefully removed overly general statements and reduced flowery or poetic phrasing.

REVIEWER 1

Comment 1.1: *This review tries to do too much - by trying to bridge multiple processes that are intracellular (mitochondria - nucleus), or systemic (across tissues and organs), and then relate this to metabopathies that affect epigenetic states in cells and across tissues. By doing so - a clear and coherent message does not emerge. There are many interesting ideas within this review, and each could do with a more detailed extraposition and attempt to synthesise. However, in its present form - this review does not synthesise ideas, and instead 'throws up' multiple ideas, none of which is fleshed out well, nor are their connections made clear.*

Response: We thank the reviewer for his/her thoughtful and constructive criticism. We appreciate the valuable suggestions, which have helped us improve the organization, focus and flow of the manuscript. We have refined the narrative and restructured it to establish a clear flow from intracellular mechanisms to systemic regulation, highlighting the key points of mechanistic convergence. Furthermore, we have reduced topics that were insufficiently developed and added new synthesis paragraphs to help readers understand how the various processes fit within a coherent conceptual framework. We believe these revisions have significantly improved the clarity and cohesiveness of the review while preserving its interdisciplinary scope. To enhance clarity and emphasize the relationships among topics, we have introduced a hierarchical section numbering system (e.g., 1, 1.1, 1.2, etc.), which can be modified as needed to comply with EMBO Reports formatting requirements. This revised structure will help guide the reader through the progression of concepts and emphasize the ordered relationships among the processes discussed.

The manuscript now begins with the fundamental role of mitochondria in genome surveillance, including the regulation of dNTP availability and DNA repair processes. This provides a mechanistic foundation for the subsequent discussion on reciprocal communication between

mitochondria and chromatin, highlighting how nuclear and mitochondrial functions are dynamically interconnected through anterograde and retrograde signaling pathways.

Building upon this, we examine how specific metabolic pathways and metabolites act as mediators between cellular metabolism and epigenetic regulation. The sections on one-carbon metabolism, TCA cycle intermediates and fatty acid metabolism are organized to illustrate how metabolic cues are translated into chromatin modifications and transcriptional responses. This progression links organelle-level communication to the broader role of metabolites in shaping epigenetic states and gene expression patterns.

Having established these cellular mechanisms, the review then expands to circulating metabolites as mediators of inter-organ communication, linking cellular mechanisms to systemic regulation. This section serves as a bridge between cellular metabolic-epigenetic interactions and organism-wide physiological regulation.

Finally, the sections on next-generation diagnostics and therapeutics, followed by the concluding perspectives, provide a forward-looking discussion of the translational implications arising from these mechanistic advances.

We hope that we have now been able to transform the manuscript from a collection of related concepts into a more integrated narrative that progresses logically from mitochondrial and chromatin biology, through metabolic-epigenetic signaling, to systemic regulation, disease mechanisms and therapeutic opportunities.

Comment 1.2: *There are parts of this review that is excellent, and interesting, but this does not come together. In addition, there are some assumptions made that are somewhat incorrect. So, while this raises interesting ideas, it is hard to figure out what the end message is. The part that is more coherent is the highlighting that the mitochondria and nuclear function are deeply coupled and integrated, as well as the relationship between the mitochondria, dNTP pools and genome stability. This part (and mito - nuclear cross talk) could become the cornerstone of this review.*

Response: We thank the reviewer for this insightful comment. We appreciate the recognition of the sections discussing mitochondrial-nuclear crosstalk and the role of mitochondria in regulating dNTP pools and genome stability. We have revised our manuscript to strengthen this narrative and improve the integration of the different topics discussed. The sections on mitochondrial control of dNTP supply, DNA repair and bidirectional mitochondria-chromatin communication establish the core framework of the manuscript (**Section 2:** Mitochondria in Genome Surveillance, beyond Powerhouse Duties, **Section 3.** Reciprocal Rewiring of Chromatin and Mitochondria, and **Section 4.** Metabolites inscribe stress signals into the epigenome).

Subsequent sections on systemic metabolic signalling and epimetabopathies are presented as extensions of the fundamental mitochondria-nucleus crosstalk to higher levels of biological organization and disease relevance. These latter sections have also benefited from constructive feedback from other reviewers, which helped refine their clarity and presentation.

Comment 1.3: *on mitochondrial TCA intermediate as substrates for acetyltransferases/demethylases - this is an over simplistic statement. The pools of mitochondrial acetylCoA and nucleo-cytosolic AcCoA are distinct, partitioned, and obtained differently (for example, much of the nuclear acetylation comes from AcetylCoA sourced from cytosolic acetate) etc.*

Response: We thank the reviewer for highlighting the need to more accurately represent the compartmentalization and sources of different TCA cycle metabolite pools and their involvement in epigenetic regulation. To address this concern, we have substantially revised Section 4.2 (TCA cycle intermediates governing gene transcription). The revised text now discusses the compartmentalization of metabolite pools and emerging evidence for local nuclear metabolic pathways. Specifically, we have expanded the discussion of nuclear acetyl-CoA generation through citrate-derived ACLY activity as well as nuclear ACSS2- and PDH-mediated production from acetate and pyruvate, respectively. We have also included evidence for the presence of TCA cycle enzymes within the nucleus that contribute to local generation of α-KG, succinate and fumarate, describing how TCA cycle-derived metabolites influence chromatin-modifying enzymes and gene regulation.

The revised section now reads:

"***4.2. TCA cycle intermediates governing gene transcription***

The mitochondria-governed metabolic-epigenetic coupling facilitated by the TCA cycle is a testimony to the fact that this pathway is not merely an energy factory. Inside the mitochondria, the TCA cycle maintains nutrient and redox balance. Additionally, several TCA cycle intermediates help control enzymes involved in chromatin modification.

This diversity of TCA cycle-derived metabolites and cofactors involved in epigenetic regulation raises an important question: how are these molecules supplied to the nucleus and what mechanisms govern their turnover after the utilization? A conventional explanation would suggest that these metabolites originate exclusively from the mitochondrial TCA cycle and are subsequently transported to the nucleus. However, this model relies on two major assumptions. First, the existence of a direct communication or sensing mechanism between mitochondria and the nucleus, and second, the presence of efficient transport systems capable of delivering these metabolites to their nuclear targets. While no definitive evidence supports a dedicated mitochondria-nuclear sensing system, transport-based delivery of metabolic intermediates to the nucleus would likely be energetically expensive. Given that epigenetic regulation is highly dynamic and tightly controlled, cells must possess mechanisms that allow rapid and precisely regulated supply and removal of metabolites within the nucleus itself. This challenge becomes even more significant because cells must continuously adapt to environmental fluctuations such as nutrient availability and metabolic stress. These external cues influence essential cellular processes, including differentiation, proliferation and stress adaptation, often by rapidly remodelling chromatin structure and transcriptional programs. Such responses require a rapid and precise supply of metabolic cofactors. Beyond the canonical point of view, emerging evidence suggests that cells have evolved a more integrated metabolic architecture involving non-canonical and nuclear TCA cycle pathways (Li et al, 2022; Arnold et al, 2022). These interconnected systems function to bridge mitochondrial metabolism with nuclear chromatin regulation, ensuring localized and timely generation or clearance of epigenetically relevant metabolites.

For example, acetyl-CoA, a central metabolite of several interconnected metabolic pathways, serves as a key substrate for histone acetylation. It can be produced within the mitochondria from pyruvate, fatty acids β-oxidation, certain amino acids or acetate. Mitochondrial citrate synthase subsequently condenses acetyl-CoA with oxaloacetate to form citrate. This citrate can be exported to the cytosol by the Solute Carrier Family 25 Member 1 (SLC25A1) transporter and is further translocated into the nucleus, where nuclear ATP-citrate lyase (ACLY) reconverts it into acetyl-CoA and oxaloacetate (Wellen et al, 2009). In addition, nuclear Acyl-CoA Synthetase Short Chain Family Member 2 (ACSS2) and Pyruvate Dehydrogenase (PDH) further enrich the nuclear acetyl-CoA pool by utilizing acetate and pyruvate, respectively (Pietrocola et al, 2015; Wiese & Bannister, 2020; Sutendra et al, 2014). Thus, the presence of TCA cycle enzymes inside the nucleus feeds the epigenetic regulators in maintaining chromatin architecture. Further, the acetyl-CoA to CoA ratio links metabolism to chromatin structure and gene expression, as HATs are inhibited by their product CoA (Sivanand et al, 2018). Thus, the ratio is a direct reflection of the cellular nutrient status rather than acetyl-CoA itself. For example, in the fed state, a high acetyl-CoA/CoA ratio in the nucleus promotes histone acetylation and open chromatin, supporting gene expression. Conversely, in the fasted state, lower acetyl-CoA favours histone deacetylation, causing chromatin compaction (Shi & Tu, 2015).

Beyond histone acetylation, several other epigenetic modifications, including histone succinylation, histone methylation and DNA methylation, also depend on TCA cycle-derived metabolites. For example, members of the amine oxidase family of histone demethylases, such as LSD1, require FAD^{+} as a cofactor to mediate proton transfer during demethylation (Shi et al, 2004). In contrast, Jumonji-domain-containing histone demethylases depend on α-ketoglutarate (α-KG) as a co-substrate, converting α-KG to succinate during catalysis (Tsukada et al, 2006). A similar requirement exists in DNA demethylation, where TET dioxygenases utilize α-KG to oxidize 5-methylcytosine (5mC) sequentially into 5-hydroxymethylcytosine (5hmC), 5-formylcytosine (5fC) and 5-carboxylcytosine (5caC) (Wu & Zhang, 2017). More than sixty α-KG-dependent dioxygenases have been identified in mammalian cells, the majority of which function within the nucleus (Rose et al, 2011). The functioning of these α-KG-dependent dioxygenases depends largely on the balance between α-KG and competitive inhibitory metabolites like succinate and fumarate from the TCA cycle or 2-hydroxyglutarate (2-HG), a α-KG-derived metabolite (Baksh & Finley, 2021; Xiao et al, 2012; Sciacovelli et al, 2016; Martínez-Reyes & Chandel, 2020). The presence of essential TCA cycle enzymes, like citrate synthase (CS), aconitase 2 (ACO2), isocitrate dehydrogenase 3 (IDH3) and oxoglutarate dehydrogenase (OGDH), within the nucleus maintains the production of α-KG, succinate and fumarate from the nuclear citrate pool (Nagaraj et al, 2017; Wang et al, 2017). Thus, metabolite ratios such as acetyl-CoA/CoA and α-KG/succinate or fumarate determine the chromatin state and transcriptional outcome.

Taken together, the mitochondrial metabolites-mediated regulation of chromatin architecture is a well-established phenomenon. The fluctuations in the abundance of such metabolites are translated into locus-specific chromatin changes, rather than bringing about genome-wide alterations. These precise transcriptional programs are modulated by various epigenetic regulatory proteins (Cao et al, 2025; Mondal et al, 2026). However, the precise mechanism by which these epigenetic regulators exert their functions remains an active area of investigation.”

Comment 1.4: *The connection between DNA repair and mitochondria is presented vaguely - and cause/consequence connections aren't well fleshed out or thought through. This, if tightened up, can become a very interesting segment.*

Response: We thank the reviewer for this critical input. We have revised section 2.2 (Tuning of DNA repair enzymes by Mitochondria) to better define the relationship between DNA repair and mitochondrial function, with particular attention to clarifying the causal and consequential links between these processes. The revised text incorporates a more detailed and coherent discussion as follows:

"*When stressed with genotoxic agents, our cells employ a remarkable defence system known as the DNA damage response (DDR) (Zhou & Elledge, 2000; Jackson & Bartek, 2009). Like an elite emergency squad, the DDR rapidly detects the site of injury, halts the cell cycle to prevent further mistakes and recruits an arsenal of repair enzymes to fix the problem. Such enzymes are dynamically tweaked by the mitochondria by integrating cellular metabolism with genome maintenance.*

Emerging evidence positions mitochondria as upstream regulators of the repair processes by coupling cellular metabolic state to nuclear genome maintenance. For example, during oxidative phosphorylation, mitochondria generate ATP along with the potent damaging agents, reactive oxygen species (ROS), thereby influencing DNA integrity and the energetic and redox conditions required for DNA repair (Nissanka & Moraes, 2018; Scheibye-Knudsen et al, 2015). It has been observed that impaired mitochondrial function compromises both nuclear genome stability and DDR through depletion of metabolic cofactors, like ATP, acetyl-CoA, α-ketoglutarate and NAD^+, essential for repair enzyme function (Nadalutti et al, 2022; Sykora et al, 2017; Hou et al, 2018). For example, the energy-intensive DNA repair processes and chromatin remodelling rely heavily on ATP availability. Metabolites like acetyl-CoA and α-ketoglutarate regulate chromatin accessibility and epigenetic modifications, thereby influencing DNA repair efficiency, as discussed in subsequent sections. The NAD^+ pool of the cell serves as a central node connecting mitochondrial redox state to both Poly (ADP-ribose) polymerase (PARP) -mediated DNA repair and sirtuin-dependent epigenetic regulation (Li et al, 2017; Lehmann et al, 2016; Croteau et al, 2017). The NAD^+-dependent deacetylase, SIRT1 can be identified as a metabolic-genomic rheostat that links mitochondrial functioning to nDNA repair. SIRT1 drives both mitochondrial biogenesis and enhanced DNA repair by Ku70 deacetylation, aiding non-homologous end joining of double-strand breaks. The mito-nuclear control of genome maintenance is thus reinforced by SIRT1 through its coordinated regulation of mitochondrial homeostasis and repair of nuclear double-strand DNA breaks (Majeed et al, 2021; Jeong et al, 2007).

Furthermore, the mitochondria are involved in several DNA repair disorders like ataxia-telangiectasia (AT), Cockayne syndrome (CS) and xeroderma pigmentosum (XP), to name a few (Scheibye-Knudsen et al, 2012; Valentin-Vega et al, 2012; Shiloh & Ziv, 2013; Fang et al, 2016). The disruption of mitochondrial quality control severely impairs DNA repair capacity in xeroderma pigmentosum group A (XPA), a nucleotide excision repair illness. A key mechanism linking mitochondria to DDR regulation can be exemplified by the NAD^+-SIRT1-PGC-1α axis. In XPA cells, persistent DNA damage leads to the chronic hyperactivation of Poly (ADP-ribose) Polymerase 1 (PARP-1), a crucial NAD^+-consuming enzyme involved in DDR. Depletion in NAD^+ results in impaired SIRT1-dependent deacetylation, attenuating the downstream Peroxisome

proliferator-activated receptor gamma coactivator 1-alpha (PGC-1α) pathway, ultimately compromising DNA repair capacity in XPA cells. This decreased activity of the NAD^+-SIRT1-PGC-1α axis can be corrected by supplementation with NAD^+ precursors (Fang et al, 2014; Verdin, 2015; Cantó et al, 2015; Bai et al, 2011).

Likewise, mitochondrial stress influences the mito-nuclear communication, explicitly linking this to DNA repair processes. mtDNA stressors and inhibitors of mitochondrial metabolism trigger retrograde calcium signalling (Biswas et al, 1999). The released Ca^{2+} ions from the mitochondria result in reduced levels of nuclear factor-κB (NF-κB), a factor directly linked to DDR and post-translational modification of key repair proteins (Biswas et al, 1999; McCool & Miyamoto, 2012; Janssens & Tschopp, 2006). These findings, linking mtDNA stress and nuclear transcription, indicate that mitochondrial dysfunction, through disrupted Ca^{2+} signalling, can influence the efficiency of DDR mechanisms.

Notably, mitochondria, the major producers of cellular ATP, fine-tune replication fidelity and resilience to stress via spatiotemporally controlled energy relocation. Mitochondrial positioning can regulate local ATP availability near the nucleus, particularly under mechanical confinement, where increased nucleus-mitochondria proximity generates a localized nuclear ATP surge that supports efficient repair and cell cycle progression (Ghose et al, 2025). Thus, spatial redistribution of mitochondria offers an additional layer of control over nuclear repair dynamics by modulating energy supply.

The mitochondrial structure and function are other critical determinants of nuclear DDR. Genotoxic assault usually promotes transient mitochondrial hyperfusion to mitigate the stress. Loss of fusion by MFN1/ MFN2 disruption results in fragmented mitochondria, dampened ATM signalling and failure in the activation of the JNK signalling pathway. Following this, there is a reduction in BRCA1 and 53BP1 foci formation, compromising both homologous recombination and non-homologous end-joining mediated DNA damage repair (Oanh et al, 2022)."

To effectively summarise this section, we have added the following:
"*Collectively, these findings support a model in which mitochondrial metabolic capacity, signalling, spatial organization, morphology and dynamics act in concert to regulate DDR efficiency through coordinated control of cellular energy state, redox balance and activation of DNA repair pathways.*"

Comment 1.5a: *This cause/consequence confusion becomes more apparent in the current presentation of the nuclear-chromatin/mitochondrial cross-control (anterograde/retrograde). It is obvious that global gene expression state will respond to mitochondrial states, and vice versa - this is essential for cellular homeostasis, or a change in state. This will include components of redox, or co-factor depletion or ATP, or calcium etc.*

Response: We thank the reviewer for highlighting the potential cause-consequence ambiguity in our original presentation of nuclear-mitochondrial crosstalk. To address this concern, we have revised and reorganized Section 3 (Reciprocal Rewiring of Chromatin and Mitochondria) to emphasise the reciprocal nature of mito-nuclear communication and the interdependence of

anterograde and retrograde signalling pathways. Specifically, we now introduce the section by stating:
"*The orchestrated mito-nuclear communication operates bidirectionally via intricate anterograde and retrograde signalling, essential for integrating the metabolic and genomic demands of the cell. The nuclear anterograde signalling exerts mitochondrial control by regulating nuclear-encoded mitochondrial gene expression. Conversely, the mitochondria relay information back to the nucleus through retrograde signals to regulate transcriptional and epigenetic reprogramming. The persistent molecular crosstalk between these two complementary axes is essential for maintaining organellar health."*
To further clarify how nuclear and mitochondrial states mutually influence one another, we note:
"*The nuclear chromatin state directly or indirectly governs the mitochondrial metabolite levels of acetyl-CoA, α-ketoglutarate, NAD^+ and S-adenosylmethionine, which afterwards serve as essential cofactors or donors for chromatin-modifying enzymes.*"
We discuss mitochondrial metabolites, including acetyl-CoA, α-ketoglutarate, succinate, fumarate and NAD^+ , as regulators of chromatin-modifying enzymes, as well as mitochondria-derived reactive oxygen species (mtROS) as redox-sensitive signalling mediators. Finally, to directly address the concern regarding cause-consequence interpretation, we have added the following concluding paragraph in the later section (4. Metabolites inscribe stress signals into the epigenome):
"*Thus, mitochondrial metabolic intermediates, with the help of epigenetic regulators, can regulate the chromatin architecture and gene transcription pattern in a spatiotemporal manner. Similarly, the nuclear genome regulates the availability and abundance of epigenetically relevant mitochondrial metabolite pools by transcribing the crucial enzymes responsible for their production. Therefore, rather than being straightforward linear cause-and-effect mechanisms, the anterograde or retrograde pathways form complementary, interdependent entities to maintain cellular integrity and homeostasis.*"

We have reorganized Section 3, "Reciprocal Rewiring of Chromatin and Mitochondria", with two subsections: Section 3.1, "Chromatin shapes mitochondrial function: the anterograde control", and Section 3.2, "Mitochondria rewrites chromatin: the retrograde feedback". We have introduced these subsections to systematically discuss the distinct yet functionally interdependent mechanisms underlying bidirectional mito-nuclear communication.

We believe these revisions clarify that mito-nuclear communication is bidirectional and operates through interconnected anterograde and retrograde signalling pathways that jointly integrate metabolic and genomic demands to maintain cellular homeostasis.

Comment 1.5b: *The fact that mitochondria are dynamic signalling hubs is now very clear. So it isn't very clear what the authors want to emphasise on. When suddenly statements like "The mitochondria thereby work as epigenomic sculptors, imprinting stress into the chromatin as sturdy, durable epigenetic marks through a cascade of transient metabolic signalling." It is totally unclear how, or what the specific mechanisms are, and how this is related to future stress responses.*

Response: We thank the reviewer for his/her comment and understand the concern regarding the mechanistic basis of the statement. We have revised the sentence to improve clarity which now

reads:
"*By modulating the availability of metabolites that serve as substrates, cofactors or inhibitors of chromatin-modifying enzymes, mitochondria translate transient stress-induced metabolic changes into alterations in histone and DNA modification patterns.*"
This revised statement introduces the theme of Section 4 (Metabolites inscribe stress signals into the epigenome), that discusses how mitochondrial stress-induced metabolic alterations can influence the epigenetic landscape. The specific mechanisms underlying this process are subsequently discussed in detail in Sections 4.1 (One-carbon metabolism-mediated chromatin methylation), 4.2 (TCA cycle intermediates governing gene transcription) and 4.3 (Fatty acid metabolism mediates histone modification). We have kept these subsections together under the broader section titled "Metabolites inscribe stress signals into the epigenome" to provide a mechanistic framework explaining how mitochondrial metabolites regulate chromatin modifications and thereby influence transcriptional responses to stress. In addition, Sections 4.1 to 4.3 have been substantially revised in response to the reviewers' comments, with expanded mechanistic clarifications to clearly illustrate how specific mitochondrial metabolites influence epigenetic enzymes, chromatin states and gene expression.

Comment 1.6: *The transition to SAM and SAM metabolism, which is of course closely coupled to 1C-folate metabolism, is unclear. What specific point do the authors want to make, related to SAM metabolism, mitochondria and nuclear/chromatin cross talk.*

Response: We thank the reviewer for his/her comment. We have revised the section to state the connection between mitochondrial function, SAM metabolism and the nucleus. Specifically, we now highlight that mitochondria provide the formate flux necessary for the cytosolic methionine cycle. The link between mitochondrial activity and the nuclear chromatin landscape is governed by the compartmentalization of 1C metabolism. The revised section now reads:
"*Mitochondria serve as a primary source of 1C units that are derived from serine catabolism via Serine hydroxymethyltransferase 2 (SHMT2), a mitochondrial enzyme. These 1C units are exported to the cytosol as formate to fuel the methionine cycle and S-adenosylmethionine (SAM) production (Ducker & Rabinowitz, 2017). SAM is the universal methyl group donor for DNA, RNA and histone methylation, regulating gene expression.*"
We further clarify that the 'crosstalk' occurs because histone methyltransferases act as metabolic sensors, where their affinity for SAM allows the chromatin state to respond dynamically to mitochondrial 1C output.

Comment 1.7: *The relationship between succinate, fumarate, 2-OG and DNA/histone demethylases is very well known. But it is still unclear how changes in these pools of metabolites can lead to specific changes in patterns of gene expression. This is a widely contested area of research, where there are large gaps in quantitative information that is necessary. While I appreciate the idea, it is not clear what point can be made.*

Response: We thank the reviewer for his/her comment. We agree that although the regulation of α-KG-dependent dioxygenases by α-KG, succinate, fumarate and 2-HG is well established, the precise mechanisms by which fluctuations in these metabolites generate locus-specific gene expression programs remain incompletely understood.

In response, we have substantially revised Section 4.2 (TCA cycle intermediates governing gene transcription) to focus on the established mechanistic links between TCA cycle-derived metabolites and chromatin-modifying enzymes. In particular, we have highlighted emerging evidence for non-canonical nuclear TCA cycle pathways and compartmentalized nuclear metabolite pools, which may facilitate the localized generation and turnover of epigenetically relevant metabolites. We have also moderated our conclusions and avoided implying a deterministic relationship between metabolite abundance and specific transcriptional outputs. The revised text emphasizes that TCA cycle metabolites influence chromatin-modifying enzymes and chromatin states, while acknowledging that the quantitative basis and gene-specific consequences of these metabolic changes are yet to be determined. However, in the context of locus-specific alteration of histone and DNA modification in a spatiotemporal manner, epigenetic regulators like writer, reader and eraser proteins play an important role. In a particular condition, the epigenetic reader proteins recruit certain epigenetic writer or eraser proteins to a specific region of the genome and regulate gene transcription in a cellular state-dependent manner. Unfortunately, the interplay of epigenetic regulators in the regulation of metabolite-mediated transcriptional regulation is beyond the scope of this review. Yet, we have added the following conclusion in the revised text: "*Taken together, the mitochondrial metabolites-mediated regulation of chromatin architecture is a well-established phenomenon. The fluctuations in the abundance of such metabolites are translated into locus-specific chromatin changes, rather than bringing about genome-wide alterations. These precise transcriptional programs are modulated by various epigenetic regulatory proteins (Cao et al, 2025; Mondal et al, 2026). However, the precise mechanism by which these epigenetic regulators exert their functions remains an active area of investigation.*"

Comment 1.8: *The links between fatty acid metabolism and histone modifications can be better fleshed out - to make a synthesising point. There is substantial, and growing literature in this space - particularly how this affects acetylation/succnylation/butyryltion programs etc. This section, if fleshed out well - in a more coherent reorganisation, can help bring this review together.*

Response: We thank the reviewer for this valuable suggestion. We have substantially revised this section to provide a more integrated discussion of how fatty acid metabolism regulates the epigenome by altering acyl-CoA metabolite availability. The revised text now emphasizes the mechanistic connection between fatty acid-derived metabolites, their incorporation into histones and the resulting effects on chromatin accessibility and transcriptional regulation. We have expanded the discussion of acetylation, succinylation, butyrylation and other emerging histone acyl modifications, while incorporating the roles of acyltransferases, deacylases and non-enzymatic acylation mechanisms to provide a more cohesive view of metabolic control of epigenetic regulation. The revised section reads:

"*Apart from all the above-mentioned traditional chromatin modifications, emerging studies describe the role of several new histone acylation modifications and their role in transcriptional regulation. Beyond their conventional role as energy sources, free fatty acids (FFAs) contribute substantially to cellular pools of acetyl-CoA, propionyl-CoA, butyryl-CoA, crotonyl-CoA, succinyl-CoA and other acyl-CoA intermediates. Fluctuations in the abundance of these metabolites provide a direct mechanism through which cellular metabolic status can influence chromatin structure and transcriptional activity.*

Among the various modifications named above, histone acetylation by acetyl-CoA from the

cellular acetate pool is most widely characterised. However, other fatty acid-derived metabolites can also facilitate a range of histone acylations, like propionylation, butyrylation, crotonylation and succinylation (Nshanian et al, 2025; Li et al, 2016; Papsdorf & Brunet, 2019). Short-chain fatty acids, particularly butyrate and propionate, are vital mediators of metabolic-epigenetic communication. These metabolites influence gene regulation by serving as precursors for histone acyl modifications, namely butyrylation and propionylation, while also modulating histone deacetylase activity. For instance, butyrate acts as an inhibitor of histone deacetylases (HDACs), increasing histone acetylation and enhancing butyrylation-driven gene activation (Donohoe et al, 2012). Succinylation of histone lysine residues, driven by succinyl-CoA, represents another fatty acid metabolism-linked modification of considerable functional importance. It is enriched at transcriptional start sites and active genomic regions, where its presence correlates with increased gene expression, suggesting a role in promoting transcriptional activity (Li et al, 2023; Liu et al, 2021).

Several histone acetyltransferases (HATs) have recently been identified to transfer a variety of acyl groups to the lysine residues on histones. For example, p300 promotes crotonylation, butyrylation, glutarylation and succinylation, albeit it is typically identified for acetylation (Goudarzi et al, 2016; Sabari et al, 2015; Kaczmarska et al, 2017). Surprisingly, relative to its acetyltransferase activity, the crotonyltransferase role of p300 exerts a much more potent stimulatory effect on transcription, enhancing gene expression (Sabari et al, 2015). Similarly, histone propionylation can be catalysed by the HATs Gcn5 and p300-CBP-associated factor (PCAF) (Berndsen et al, 2007; Leemhuis et al, 2008).

The dynamicity of the histone acylation marks is further regulated by enzymes that remove these modifications. For example, in addition to their well-established deacetylase function, the NAD^+-dependent sirtuins, SIRT1, SIRT2 and SIRT3 have been identified as histone decrotonylases (Bao et al, 2014). Several other modifications like malonylation, 2-hydroxyisobutyrylation and β-hydroxybutyrylation, further expand the repertoire of histone marks that respond to changes in fatty acid metabolism. Acylation of histones can also occur by non-enzymatic covalent modifications (NECMs), bypassing the conventional epigenetic "writer" proteins. The elevated intracellular concentration and intrinsic chemical reactivity of the acyl-CoA metabolites help them bind with histone lysine residues spontaneously (Maksimovic & David, 2021; Diehl & Muir, 2020). Thus, alterations in fatty acid metabolism can influence chromatin states through both enzymatic and non-enzymatic mechanisms. Together, these emerging histone acyl modifications flawlessly link fatty acid metabolism to transcriptional regulation."

Comment 1.9: *the section on circulating metabolites is a conceptual jump - it is very difficult to specifically link mitochondrial balance to systemic changes (after circulation) and from here move to what the authors describe as 'epimetabopathies'. From here, making a jump to phase-transitioning and phase separation, and chromatin/gene expression changes appears completely disjointed - and an enthusiastic expression of multiple possible ideas, but not a rigorous exposition.*

Response: We thank the reviewer for this observation. We agree that the original version did not sufficiently explain the conceptual progression from mitochondrial metabolic homeostasis to circulating metabolites, systemic signalling and ultimately epimetabopathies. To address this

concern, we have substantially revised the transition between these sections and have explicitly acknowledged the current limitations of the field.

First, to establish a mechanistic link between intracellular mitochondrial metabolism and systemic metabolite signalling, we added the following text:
"*Further, the compartmentalised mitochondria-derived cellular metabolic pools are not restrained within the boundaries of the cell. During several physiological assaults, these metabolites can be secreted from cells into the extracellular milieu, thereby acting as signalling molecules or biomarkers for specific pathophysiological consequences.*"

Second, we expanded the discussion of circulating metabolites by incorporating specific examples of metabolite-mediated inter-organ communication, including acetate-dependent liver-brain signalling, MCT1-mediated metabolic crosstalk and circulating mitochondrial N-formyl peptides. These examples were included to provide concrete evidence that alterations in metabolic states can influence distant tissues through circulating metabolic signals.

Third, to avoid overstating current knowledge and to acknowledge the limitations highlighted by the reviewer, we write:
"*Systemic metabolite signalling is an emerging field and much remains to be discovered. The mechanisms underlying how metabolites signal between tissues and how they can be targeted in disease are still poorly understood.*"

Finally, we would like to clarify that the concept of "epimetabopathies" is introduced as a framework for understanding how systemic metabolic perturbations may influence epigenetic proteins, rather than as a direct consequence of all circulating metabolic changes. The revised text now presents these topics as a continuum of hypotheses and evidence with varying degrees of experimental support, rather than as a single established mechanistic pathway. We have also added transitional text to make clear that the links between systemic metabolism and "epimetabopathies" remain areas of active investigation.
"*While circulating metabolites serve as mediators of inter-organ communication, perturbations in this delicate metabolic signalling cascade can have profound pathological consequences. The capacity of the circulating metabolites to bridge distant organ systems underscores a deeper, intrinsic dependency of the cellular machinery on metabolic fluctuating states. As described previously, the metabolites connect mitochondrial function with the epigenome, shaping gene expression patterns. This local, bidirectional communication when disrupted, may result in disease phenotypes. This may be the effect of metabolic dysfunction, altered metabolite levels or mutations in epigenetic regulators. Studying these intracellular and intercellular networks uncovers the molecular basis of 'epimetabopathies,' a group of disorders where defects in epigenetic-metabolic crosstalk serve as the primary driver of several ailments.*"

Comment 1.10: *On the figures - Figure 1 is terrific and comprehensive. Figure 3 is euphemistic - and impossible to present as established fact.*

Response: We thank the reviewer for his/her comment. We agree that Figure 3 is intended as a conceptual framework highlighting proposed mechanisms of metabolite-mediated interorgan crosstalk based on the sparse existing literature, rather than established causal relationships. To

avoid overstating the current evidence, we have revised the figure legend throughout to use more cautious language and explicitly indicate that several of the depicted pathways and epigenetic modifications are hypothetical, emerging or remain to be experimentally validated in the context of liver-brain communication. The revised Figure 3 and its corresponding legend now read:

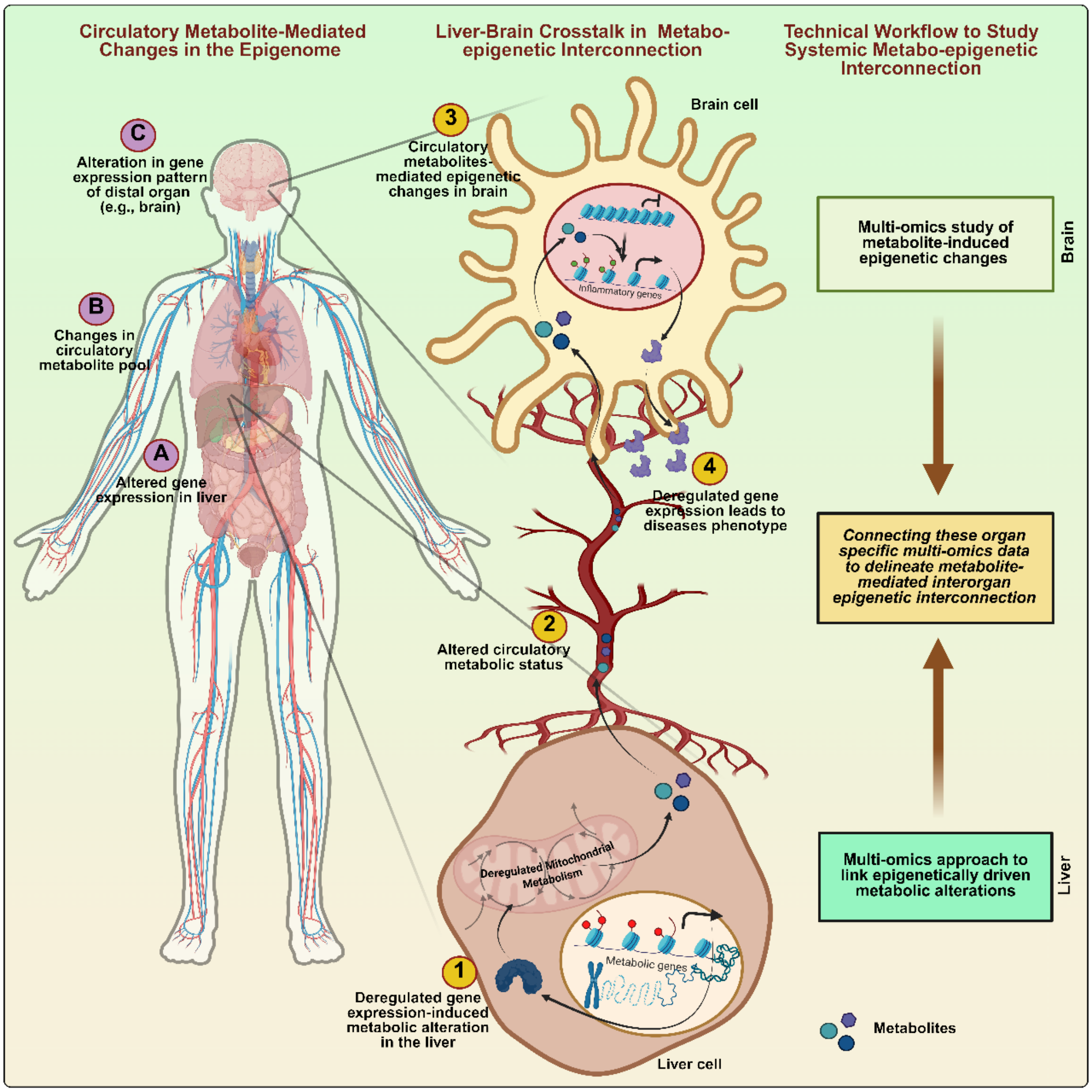

"***Figure 3. Proposed model of metabolite-mediated interorgan crosstalk and its potential contribution to epigenetic and metabolic reprogramming across the liver-brain axis.*** *Alterations in hepatic gene expression, which may occur under different dietary or metabolic conditions, can reshape the hepatic metabolic landscape and potentially alter the pool of circulating metabolites. These metabolic intermediates may reach distal organs like the brain, where they could cause epigenetic rewiring and changes in gene expression patterns, thereby contributing to disease-associated phenotypes. The left panel (A - C) illustrates putative systemic effects, while the middle panel (1 - 4) depicts proposed intercellular mechanisms. The epigenetic modifications depicted may regulate gene expression, but are yet to be explored in the context of metabolite-mediated interorgan crosstalk. The right panel highlights the proposed multi-omics approach that may facilitate the mapping of organ-specific epimetabolic states and help evaluate these inter-organ*

communication pathways. Created in BioRender. Das, C. (2026) https://BioRender.com/l3x7x4r."

REVIEWER 2

Comment 2.1: *This manuscript reviews a complex, yet very relevant topic: the cross-talk between mitochondrial metabolism and nuclear epigenetic processes. The manuscript first deals with the regulation of the nucleotide pools that serve both nuclear and mitochondrial DNA synthesis. Imbalance in one the two processes inevitably affects the other. This has profound effects on the ability of cells to repair DNA that is in turn related to cell cycle control and cell fate.*

Response: We thank the reviewer for his/her thoughtful summary of our work.

Comment 2.2: *Here, the manuscript discusses the role of SIRT1. The narrative mentions "SIRT1's synchronous activity". At this point, I was a bit lost as the meaning of "synchronous" in this context is not fully clear. A better explanation will help the reader.*

Response: We thank the reviewer for pointing this out. To improve clarity, we have revised the sentence to state that SIRT1 coordinately regulates mitochondrial biogenesis and nuclear DNA repair pathways. The revised text now reads:
"*SIRT1 drives both mitochondrial biogenesis and enhanced DNA repair by Ku70 deacetylation, aiding non-homologous end joining of double-strand breaks. The mito-nuclear control of genome maintenance is thus reinforced by SIRT1 through its coordinated regulation of mitochondrial homeostasis and repair of nuclear double-strand DNA breaks.*"

Comment 2.3: *The manuscript continues by discussing anterograde and retrograde signallings. Anterograde signaling is mostly the regulated expression and function of transcription factors that control the expression of mitochondrial proteins. Retrograde signalling alerts the nucleus of a stressful condition of mitochondria.*

Response: We thank the reviewer for this concise summary of the sections on anterograde and retrograde signalling.

Comment 2.4: *Here, the text mentions a change in H3K9 methylation. It would be interesting to describe, if known, how such a change is brought about and why it is specific for this methylation mark. This is the core of the review because one appreciates how mitochondrial metabolites can directly affect the activity of chromatin-modifying enzymes. Indeed, alterations in these processes are tumorigenic.*

Response: We thank the reviewer for this insightful comment. We agree that elaborating on how this chromatin change is brought about would strengthen the review considerably. We have not included these details in the original text in the interest of maintaining focus and adhering to the journal's word limit. However, we would be happy to incorporate the following explanation in the manuscript if the reviewers and the editor consider this level of mechanistic detail appropriate for the scope of the review:

"*Mechanistically, mitochondrial stress drives the nuclear accumulation of LIN-65, which works with the H3K9 methyltransferase MET-2 to catalyse genome-wide H3K9 dimethylation, causing global chromatin compaction. This reorganisation in the chromatin facilitates recruitment of the transcription factor DVE-1 to specific genomic loci, enabling activation of the mitochondrial unfolded protein response (UPRmt). Loss of either MET-2 or LIN-65 abolishes H3K9me2 accumulation and suppresses UPRmt activation, demonstrating that H3K9me2 is necessary for mitochondrial stress response.*"

Reference (previously cited in the manuscript): *Tian Y, Garcia G, Bian Q, Steffen KK, Joe L, Wolff S, Meyer BJ & Dillin A (2016) Mitochondrial Stress Induces Chromatin Reorganization to Promote Longevity and UPRmt.* ***Cell*** *165: 1197–1208*

Regarding the specificity of H3K9me2, Tian et al. have shown that the UPRmt is principally dependent on MET-2, which has an intrinsic enzymatic specificity for H3K9 mono- and dimethylation but not trimethylation. Other methyltransferases, like SET-25 are dispensable in this regard. Based on these findings, we speculate that H3K9me2 may be favoured as it facilitates a facultative, reversible heterochromatin state that supports a dynamic response to transient organelle stress.

Comment 2.5: *At this point, the review mentions N-formylpeptides entering circulation and functioning as immunomodulators. This paragraph might be more logically moved to the section on "Circulating metabolites".*

Response: We thank the reviewer for this suggestion. We agree that N-formyl peptides are discussed in the context of their release into circulation and systemic signalling. Accordingly, we have now moved this to the "Circulating Metabolites" section to improve the logical flow and organisation.

Comment 2.6: *The manuscript makes the point that alterations in these regulatory circuits can be pathogenic. The discussion of Rett syndrome is particularly illuminating in this regard. Time will tell if this knowledge can be translated into efficient pharmacological therapies.*

Response: We thank the reviewer for this assessment. We are pleased that the discussion on Rett syndrome effectively exemplifies the pathological consequences of disrupted mitochondrial-epigenetic regulatory circuits. We agree that translating these mechanistic insights into effective pharmacological therapies remains an important future challenge and opportunity for the field.

Comment 2.7: *Overall, I found this review useful and timely. Aside from the minor suggestions offered above, it can be published in this Journal.*

Response: We thank the reviewer for the positive evaluation of our manuscript and for considering the review useful and timely. We also appreciate the constructive suggestions provided, which have helped improve the clarity and overall organisation of our manuscript. We have carefully addressed all comments and revised the manuscript accordingly.

Comment 2.8: *Abstract The sentence "Mitochondrial metabolites are the main directors of life's molecular ballet" is somewhat cryptic obscure and probably overstated.*

Response: We thank the reviewer for his/her comment. We have now modified the statement as follows:
"Mitochondrial metabolites serve as vital regulators of cellular physiology"

REVIEWER 3

Comment 3.1: *In this review article, the authors describe how mitochondrial metabolites can regulate epigenetics in physiological and disease conditions. In this lyrically written manuscript, the authors introduce a new term - "epimetabopathies" to describe this phenomenon. This topic is of great biomedical significance, because mitochondrial dysfunction has been associated with numerous common as well as rare genetic diseases. Moreover, environmental and dietary factors, such as high fat diet, is known to perturb mitochondrial function and cellular epigenetic landscape. The authors have nicely organized their article in different subtopics - anterograde vs retrograde signaling, circulating metabolites regulating systemic interactions, etc.*

Response: We thank the reviewer for his/her positive and thoughtful assessment of our manuscript. We are pleased that the reviewer found this topic of broad biomedical relevance and appreciated the overall organization of the review.

Comment 3.2: *However, their introduction of new terms without defining them, use of some cryptic sentences, a lack of mechanistic description, tangential forays into irrelevant areas, and occasional errors makes it a difficult read.*

Response: We thank the reviewer for this constructive feedback. We have carefully revised the manuscript to improve its clarity and readability. We have defined newly introduced terms at their first occurrence, simplified or rephrased cryptic sentences, expanded mechanistic details where needed and removed tangential content that did not directly support the central theme of the review. We have also thoroughly edited the manuscript to correct errors and improve the logical flow.

Comment 3.3: *On page 5, the authors state that "mitochondria confiscate certain species of dNTPs". The authors can be more precise by identifying/listing the specific species of dNTPs.*

Response: We thank the reviewer for this helpful suggestion. We have revised the text to specify the dNTP species involved and replaced the term "directly confiscate" with "*selectively sequester*" for clarity. The sentence now reads:
"*Other than biosynthesis, mitochondria seem to selectively sequester certain species of dNTP, particularly dGTP, acting like nucleotide reservoirs.*"

Comment 3.4: *The section "Tuning of DNA repair enzymes by mitochondria" appears a bit superficial without detailed mechanistic insights and explanations of studies discussed. For example, the sentence on page 7 "It has been observed that impaired mitochondrial function compromises both nuclear genome stability and DDR through depletion of metabolic cofactors essential for repair enzyme function (Nadalutti et al, 2022; Sykora et al, 2017; Hou et al, 2018)" the authors should elaborate on which cofactors are they talking about.*

Response: We thank the reviewer for this insightful comment. We have substantially revised and

reorganized Section 2.2 (Tuning of DNA repair enzymes by Mitochondria) to provide more mechanistic details of the studies described. Regarding the metabolic cofactors involved in mitochondrial regulation of DNA repair and genome stability, we have revised this section as follows:
"*It has been observed that impaired mitochondrial function compromises both nuclear genome stability and DDR through depletion of metabolic cofactors, like ATP, acetyl-CoA, α-ketoglutarate and NAD⁺, essential for repair enzyme function (Nadalutti et al, 2022; Sykora et al, 2017; Hou et al, 2018). For example, the energy-intensive DNA repair processes and chromatin remodelling rely heavily on ATP availability. Metabolites like acetyl-CoA and α-ketoglutarate regulate chromatin accessibility and epigenetic modifications, thereby influencing DNA repair efficiency, as discussed in subsequent sections. The NAD⁺ pool of the cell serves as a central node connecting mitochondrial redox state to both Poly (ADP-ribose) polymerase (PARP) -mediated DNA repair and sirtuin-dependent epigenetic regulation (Li et al, 2017; Lehmann et al, 2016; Croteau et al, 2017).*"

Comment 3.5: *Again, on page 7, the authors should explain on how NAD+ connects to PARP?*

Response: We thank the reviewer for this suggestion. We have revised the manuscript to clarify the mechanistic link between NAD⁺ and PARP-1 as follows:
"*A key mechanism linking mitochondria to DDR regulation can be exemplified by the NAD⁺-SIRT1-PGC-1α axis. In XPA cells, persistent DNA damage leads to the chronic hyperactivation of Poly (ADP-ribose) Polymerase 1 (PARP-1), a crucial NAD⁺-consuming enzyme involved in DDR. Depletion in NAD⁺ results in impaired SIRT1-dependent deacetylation, attenuating the downstream Peroxisome proliferator-activated receptor gamma coactivator 1-alpha (PGC-1α) pathway, ultimately compromising DNA repair capacity in XPA cells.*"

Comment 3.6: *The authors should describe how mitochondrial dysfunction perturbs NAD+ homeostasis, which in turn impacts epigenetic landscape.*

Response: We thank the reviewer for his/her comment. We have revised Section 2.2 (Tuning of DNA repair enzymes by Mitochondria) to explicitly discuss NAD⁺ as a key mediator linking mitochondrial function to epigenetic regulation. The revised text now describes how mitochondrial dysfunction can perturb NAD⁺ homeostasis, thereby affecting the activity of NAD⁺-dependent sirtuins such as SIRT1, altering histone acetylation states and influencing transcriptional regulation in addition to DNA repair processes. The text now reads:
"*The NAD⁺-dependent deacetylase, SIRT1 can be identified as a metabolic-genomic rheostat that links mitochondrial functioning to nDNA repair. SIRT1 drives both mitochondrial biogenesis and enhanced DNA repair by Ku70 deacetylation, aiding non-homologous end joining of double-strand breaks. The mito-nuclear control of genome maintenance is thus reinforced by SIRT1 through its coordinated regulation of mitochondrial homeostasis and repair of nuclear double-strand DNA breaks (Majeed et al, 2021; Jeong et al, 2007).*

The mitochondria are involved in several DNA repair disorders like ataxia-telangiectasia (AT), Cockayne syndrome (CS) and xeroderma pigmentosum (XP), to name a few (Scheibye-Knudsen et al, 2012; Valentin-Vega et al, 2012; Shiloh & Ziv, 2013; Fang et al, 2016). The disruption of mitochondrial quality control severely impairs DNA repair capacity in xeroderma pigmentosum

group A (XPA), a nucleotide excision repair illness. A key mechanism linking mitochondria to DDR regulation can be exemplified by the NAD^+-SIRT1-PGC-1α axis. In XPA cells, persistent DNA damage leads to the chronic hyperactivation of Poly (ADP-ribose) Polymerase 1 (PARP-1), a crucial NAD^+-consuming enzyme involved in DDR. Depletion in NAD^+ results in impaired SIRT1-dependent deacetylation, attenuating the downstream Peroxisome proliferator-activated receptor gamma coactivator 1-alpha (PGC-1α) pathway, ultimately compromising DNA repair capacity in XPA cells. This decreased activity of the NAD^+-SIRT1-PGC-1α axis can be corrected by supplementation with NAD^+ precursors (Fang et al, 2014; Verdin, 2015; Cantó et al, 2015; Bai et al, 2011)."

In addition, we discuss the role of NAD^+ in linking mitochondrial metabolism to chromatin regulation in Section 3.2 (Mitochondria rewrites chromatin: the retrograde feedback):
"*Similarly, the critical TCA cycle electron acceptor and coenzyme, NAD^+ is the fuel for sirtuin deacetylases and poly (ADP-ribose) polymerase (PARP) enzymes involved in DDR (Verdin, 2015; Bai & Cantó, 2012; Imai & Guarente, 2014).*"

<u>Comment 3.7:</u> *The term "morphofunctional" or "dual-axis control" is confusing. It is best to avoid introducing jargons to ensure that the main message is not lost to the readers.*

Response: We thank the reviewer for his/her comment. We have replaced *"morphofunctional"* throughout the manuscript with clearer expressions like *"structural integrity and functional state"* or *"architectural and functional state"*, as appropriate to the context.

To improve clarity, we have also removed the "dual-axis control" expression and revised the text as follows:
"*A vast majority of mitochondrial proteins are encoded by the nucleus and influence mitochondrial function through two major mechanisms.*"
This explicitly describes the two major mechanisms via which nuclear-encoded factors regulate mitochondrial function, later discussed: (i) by controlling mitochondrial metabolism and homeostasis, and (ii) by regulating mitochondrial DNA replication and gene expression. Accordingly, the subsequent paragraphs have also been modified, replacing the term *"axis"* with *"mechanism"* throughout.

<u>Comment 3.8:</u> *Define acronyms on first use, for example OXPHOS, page 4. On page 9, PCG-1 should be PGC-1.*

Response: We thank the reviewer for pointing this out. We have carefully reviewed the manuscript and defined the acronyms at their first occurrence. In addition, we have added a comprehensive table of abbreviations (Table 1) to improve readability. We have also corrected the typographical error on page 9, changing PCG-1 to PGC-1.

<u>Comment 3.9:</u> *It is not clear what the author means by "chromatin substrates?" Again, it is better to spell out what they mean.*

Response: We thank the reviewer for his/her comment. By "chromatin substrates" we intended to refer to the mitochondrial metabolites that function as substrates or cofactors for chromatin-

modifying enzymes involved in epigenetic regulation. These include acetyl-CoA for histone acetyltransferases, α-ketoglutarate for dioxygenase-mediated demethylation reactions, etc. To improve clarity, we have replaced the phrase "chromatin substrates" with "*chromatin-modifying metabolites*" in the revised manuscript.

Comment 3.10: *The sentence "For example, extracellular metabolites like α-ketoglutarate (α-KG) can bind to the purinergic receptor, P2RX4, facilitating nuclear import of intracellular α-KG via SLC25A11" is very confusing, because SLC25A11 is a mitochondrial α-KG exporter and not a nuclear α-KG importer.*

Response: We thank the reviewer for pointing out this ambiguity. We have rewritten the statement as follows:
"*For example, extracellular metabolites like α-ketoglutarate (α-KG) can bind to the purinergic receptor, P2RX4 and promote the expression of SLC25A11 in the mitochondrial membrane, leading to the enrichment of the cytosolic α-KG pool. Increase in cytosolic α-KG induces its diffusion into the nucleus and acts as a cofactor for histone demethylase and activates seprina1e (serine or cysteine peptidase inhibitor, clade A, member 1e) gene transcription by facilitating histone H3K27me3 demethylation at its promoter, thus suppressing gluconeogenesis and hyperglycemia (Yuan et al, 2022).*"

Prof. Siddhesh Kamat
Indian Institute of Science Education and Research (IISER) Pune
Department of Biology
Dr. Homi Bhabha Road, Pashan
Pune, Maharashtra 411008
India

Dear Siddhesh,

I am pleased to inform you that your Review has been accepted for publication in EMBO reports. Your manuscript will be processed for publication by EMBO Press. It will be copy edited and you will receive page proofs prior to publication. Please note that you will be contacted by Springer Nature Author Services to complete licensing information.

If you have any questions, please do not hesitate to contact the Editorial Office. Thank you for your contribution to EMBO Reports.

Best regards,
Esther

Esther Schnapp, PhD
Senior Editor
EMBO reports

>>> Please note that it is EMBO Reports policy for the transcript of the editorial process (containing referee reports and your response letter) to be published as an online supplement to each paper. If you do NOT want this, you will need to inform the Editorial Office via email immediately. More information is available here: https://link.springer.com/partners/embo-press/editorial-policies#Peer%20review
